# Association of Birth Weight With Type 2 Diabetes and Glycemic Traits: A Mendelian Randomization Study

**DOI:** 10.1001/jamanetworkopen.2019.10915

**Published:** 2019-09-20

**Authors:** BIRTH-GENE (BIG) Study Working Group, Tao Huang, Tiange Wang, Yan Zheng, Christina Ellervik, Xiang Li, Meng Gao, Zhe Fang, Jin-Fang Chai, Tarun veer S. Ahluwalia, Yujie Wang, Trudy Voortman, Raymond Noordam, Alexis Frazier-Wood, Markus Scholz, Emily Sonestedt, Masato Akiyama, Rajkumar Dorajoo, Ang Zhou, Tuomas O. Kilpeläinen, Marcus E. Kleber, Sarah R. Crozier, Keith M. Godfrey, Rozenn Lemaitre, Janine F. Felix, Yuan Shi, Preeti Gupta, Chiea-Chuen Khor, Terho Lehtimäki, Carol A. Wang, Carla M. T. Tiesler, Elisabeth Thiering, Marie Standl, Peter Rzehak, Eirini Marouli, Meian He, Cécile Lecoeur, Dolores Corella, Chao-Qiang Lai, Luis A. Moreno, Niina Pitkänen, Colin A. Boreham, Tao Zhang, Seang Mei Saw, Paul M. Ridker, Mariaelisa Graff, Frank J. A. van Rooij, Andre G. Uitterlinden, Albert Hofman, Diana van Heemst, Frits R. Rosendaal, Renée de Mutsert, Ralph Burkhardt, Christina-Alexandra Schulz, Ulrika Ericson, Yoichiro Kamatani, Jian-Min Yuan, Chris Power, Torben Hansen, Thorkild I. A. Sørensen, Anne Tjønneland, Kim Overvad, Graciela Delgado, Cyrus Cooper, Luc Djousse, Fernando Rivadeneira, Karen Jameson, Wanting Zhao, Jianjun Liu, Nanette R. Lee, Olli Raitakari, Mika Kähönen, Jorma Viikari, Veit Grote, Jean-Paul Langhendries, Berthold Koletzko, Joaquin Escribano, Elvira Verduci, George Dedoussis, Caizheng Yu, Yih Chung Tham, Blanche Lim, Sing Hui Lim, Philippe Froguel, Beverley Balkau, Nadia R. Fink, Rebecca K. Vinding, Astrid Sevelsted, Hans Bisgaard, Oscar Coltell, Jean Dallongeville, Frédéric Gottrand, Katja Pahkala, Harri Niinikoski, Elina Hyppönen, Oluf Pedersen, Winfried März, Hazel Inskip, Vincent W. V. Jaddoe, Elaine Dennison, Tien Yin Wong, Charumathi Sabanayagam, E-Shyong Tai, Karen L. Mohlke, David A. Mackey, Dariusz Gruszfeld, Panagiotis Deloukas, Katherine L. Tucker, Frédéric Fumeron, Klaus Bønnelykke, Peter Rossing, Ramon Estruch, Jose M. Ordovas, Donna K. Arnett, Aline Meirhaeghe, Philippe Amouyel, Ching-Yu Cheng, Xueling Sim, Yik Ying Teo, Rob M. van Dam, Woon-Puay Koh, Marju Orho-Melander, Markus Loeffler, Michiaki Kubo, Joachim Thiery, Dennis O. Mook-Kanamori, Dariush Mozaffarian, Bruce M. Psaty, Oscar H. Franco, Tangchun Wu, Kari E. North, George Davey Smith, Jorge E. Chavarro, Daniel I. Chasman, Lu Qi

**Affiliations:** 1 Key Laboratory of Molecular Cardiovascular Sciences, Peking University, Ministry of Education, Beijing, China; 2 Department of Global Health, School of Public Health, Peking University, Beijing, China; 3 Department of Epidemiology and Biostatistics, School of Public Health, Peking University Health Science Center, Beijing, China; 4 Shanghai Institute of Endocrine and Metabolic Diseases, Rui Jin Hospital, Shanghai Jiao Tong University School of Medicine, Shanghai, China; 5 Department of Nutrition, Harvard T.H. Chan School of Public Health, Boston, Massachusetts; 6 Department of Epidemiology, School of Public Health and Tropical Medicine, Tulane University, New Orleans, Louisiana; 7 School of Life Sciences, Fudan University, Shanghai, China; 8 Department of Research and Innovation Region Zealand, Region Zealand, Denmark; 9 Division of Clinical Medicine, Faculty of Health and Medical Sciences, University of Copenhagen, Copenhagen, Denmark; 10 Department of Laboratory Medicine, Boston Children's Hospital, Boston, Massachusetts; 11 Department of Pathology, Harvard Medical School, Boston, Massachusetts; 12 Saw Swee Hock School of Public Health, National University of Singapore, Singapore; 13 Copenhagen Prospective Studies on Asthma in Childhood, Herlev and Gentofte Hospital, University of Copenhagen, Copenhagen, Denmark; 14 Steno Diabetes Center Copenhagen, Gentofte, Denmark; 15 Department of Epidemiology, University of North Carolina, Chapel Hill; 16 Department of Epidemiology, Erasmus MC University Medical Center, Rotterdam, Netherlands; 17 Section of Gerontology and Geriatrics, Department of Internal Medicine, Leiden University Medical Center, Leiden, Netherlands; 18 Children's Nutrition Research Center, Baylor College of Medicine, Houston, Texas; 19 LIFE Research Center for Civilization Diseases, University of Leipzig, Leipzig, Germany; 20 Institute for Medical Informatics, Statistics and Epidemiology, University of Leipzig, Leipzig, Germany; 21 Department of Clinical Sciences Malmö, Lund University, Malmö, Sweden; 22 RIKEN Center for Integrative Medical Sciences, Laboratory for Statistical Analysis, Yokohama, Japan; 23 Genome Institute of Singapore, Agency for Science Technology and Research, Singapore; 24 Centre for Population Health Research, School of Health Sciences, University of South Australia, Adelaide, Australia; 25 Sansom Institute of Health Research, University of South Australia, Adelaide, Australia; 26 The Novo Nordisk Foundation Center for Basic Metabolic Research, Section of Metabolic Genetics, Faculty of Health and Medical Sciences, University of Copenhagen, Copenhagen, Denmark; 27 Competence Cluster of Nutrition and Cardiovascular Health, Halle-Jena-Leipzig, Germany; 28 Institute of Nutrition, Friedrich Schiller University, Jena, Germany; 29 Vth Department of Medicine, Mannheim Medical Faculty, Heidelberg University, Mannheim, Germany; 30 MRC Lifecourse Epidemiology Unit, University of Southampton, Southampton General Hospital, Southampton, United Kingdom; 31 NIHR Southampton Biomedical Research Centre, University Hospital Southampton NHS Foundation Trust, University of Southampton, Southampton, United Kingdom; 32 Cardiovascular Health Research Institute, Department of Medicine, University of Washington, Seattle; 33 The Generation R Study Group, Erasmus MC, University Medical Center Rotterdam, Rotterdam, Netherlands; 34 Department of Epidemiology, Erasmus MC, University Medical Center Rotterdam, Rotterdam, Netherlands; 35 Department of Pediatrics, Erasmus MC, University Medical Center Rotterdam, Rotterdam, Netherlands; 36 Singapore Eye Research Institute, Singapore National Eye Centre, Singapore; 37 Department of Clinical Chemistry, Fimlab Laboratories, and Finnish Cardiovascular Research Center, Tampere, Finland; 38 Faculty of Medicine and Health Technology, Tampere University, Tampere, Finland; 39 Division of Obstetrics and Gynaecology, School of Medicine, University of Western Australia, Crawley, Western Australia, Australia; 40 Department of Obstetrics and Gynecology, School of Medicine and Public Health, The University of Newcastle, Callaghan, New South Wales, Australia; 41 Ludwig-Maximilians-University of Munich, Dr von Hauner Children's Hospital, Division of Metabolic Diseases and Nutritional Medicine, Munich, Germany; 42 Institute of Epidemiology, Helmholtz Zentrum München–German Research Center for Environmental Health, Neuherberg, Germany; 43 Division of Metabolic and Nutritional Medicine, Dr von Hauner Children's Hospital, Klinikum der Universitaet Muenchen, Munich, Germany; 44 William Harvey Research Institute, Barts and The London School of Medicine and Dentistry, Queen Mary University of London, London, United Kingdom; 45 MOE Key Lab of Environment and Health, School of Public Health, Tongji Medical College, Huazhong University of Science & Technology, Wuhan, Hubei, China; 46 University of Lille Nord de France, Lille, France; 47 Institut Pasteur de Lille, Lille, France; 48 Department of Preventive Medicine and Public Health, University of Valencia, Valencia, Spain; 49 CIBER Fisiopatología de la Obesidad y Nutrición, Instituto de Salud Carlos III, Madrid, Spain; 50 United States Department of Agriculture Research Service, Human Nutrition Research Center on Aging at Tufts University, Boston, Massachusetts; 51 Growth Exercise, Nutrition and Development Research Group, Facultad de Ciencias de la Salud, Universidad de Zaragoza, Zaragoza, Spain; 52 Research Centre of Applied and Preventive Cardiovascular Medicine, University of Turku, Turku, Finland; 53 UCD Institute for Sport & Health, University College Dublin, Dublin, Ireland; 54 Department of Biostatistics, School of Public Health, Shandong University, Jinan, China; 55 Division of Preventive Medicine, Brigham & Women's Hospital, Boston, Massachusetts; 56 Department of Internal Medicine, Erasmus MC, University Medical Center, Rotterdam, Netherlands; 57 Department of Epidemiology, Harvard School of Public Health, Boston, Massachusetts; 58 Department of Clinical Epidemiology, Leiden University Medical Center, Leiden, Netherlands; 59 Institute for Laboratory Medicine, University of Leipzig, Leipzig, Germany; 60 Division of Cancer Control and Population Sciences, UPMC Hillman Cancer Center, University of Pittsburgh, Pittsburgh, Pennsylvania; 61 Department of Epidemiology, Graduate School of Public Health, University of Pittsburgh, Pittsburgh, Pennsylvania; 62 Population, Policy and Practice, UCL Great Ormond Street Institute of Child Health, London, United Kingdom; 63 Department of Public Health, Section of Epidemiology, Faculty of Health and Medical Sciences, University of Copenhagen, Copenhagen, Denmark; 64 MRC Integrative Epidemiology Unit & School of Social and Community Medicine, University of Bristol, Bristol, United Kingdom; 65 Danish Cancer Society Research Center, Copenhagen, Denmark; 66 Department of Public Health, Section for Epidemiology, Aarhus University, Aarhus, Denmark; 67 Aalborg University Hospital, Aalborg, Denmark; 68 Oxford NIHR Musculoskeletal Biomedical Research Unit, Nuffield Department of Orthopaedics, Rheumatology and Musculoskeletal Sciences, The Botnar Research Centre, University of Oxford, Oxford, United Kingdom; 69 Department of Medicine, Brigham and Women's Hospital, Harvard Medical School, Boston, Massachusetts; 70 Department of Internal Medicine, Erasmus MC, University Medical Center Rotterdam, Rotterdam, Netherlands; 71 USC Office of Population Studies Foundation Inc, University of San Carlos, Cebu City, Philippines; 72 Department of Anthropology, Sociology, and History, University of San Carlos, Cebu City, Philippines; 73 Department of Clinical Physiology and Nuclear Medicine, Turku University Hospital, Turku, Finland; 74 Department of Clinical Physiology, Tampere University Hospital, and Finnish Cardiovascular Research Center–Tampere, Faculty of Medicine and Health Technology, Tampere University, Tampere, Finland; 75 Division of Medicine, Turku University Hospital, Turku, Finland; 76 Department of Medicine, University of Turku, Turku, Finland; 77 Department of Paediatrics and NICU, CHC-Site St-Vincent, Liège-Rocourt, Belgium; 78 Paediatrics Research Unit, Universitat Rovira i Virgili, IISPV, Reus, Spain; 79 Department of Pediatrics, San Paolo Hospital, University of Milan, Milan, Italy; 80 Department of Nutrition and Dietetics, School of Health Science and Education, Harokopio University, Athens, Greece; 81 Department of Ophthalmology, Yong Loo Lin School of Medicine, National University of Singapore, Singapore; 82 University of Lille Nord de France, Lille, France; 83 INSERM, Centre for Research in Epidemiology and Population Health, Villejuif, France; 84 University Versailles Saint-Quentin-en-Yvelines, Versailles, France; 85 University Paris Sud 11, Villejuif, France; 86 Department of Computer Languages and Systems, University Jaume I, Castellon, Spain; 87 INSERM U1167, Institut Pasteur de Lille, University of Lille, Lille, France; 88 INSERM U995, Hôpital Jeanne de Flandre, CHU-Lille, University of Lille, Lille, France; 89 Paavo Nurmi Centre, Sports and Exercise Medicine Unit, Department of Physical Activity and Health, Turku, Finland; 90 Department of Pediatrics, Turku University Hospital, Turku, Finland; 91 Department of Physiology, University of Turku, Turku, Finland; 92 Australian Centre for Precision Health, University of South Australia Cancer Research Institute, University of South Australia, Adelaide, Australia; 93 South Australian Health and Medical Research Institute, Adelaide, Australia; 94 Synlab Academy, Synlab Holding Deutschland GmbH, Mannheim, Germany; 95 Clinical Institute of Medical and Chemical Laboratory Diagnostics Medical University of Graz, Graz, Austria; 96 Victoria University of Wellington, Wellington, New Zealand; 97 Ophthalmology & Visual Sciences Academic Clinical Program (Eye ACP), Duke-NUS Medical School, Singapore; 98 Department of Medicine, Yong Loo Lin School of Medicine, National University of Singapore, Singapore; 99 Health Services and Systems Research, Duke-NUS Medical School, Singapore; 100 Department of Genetics, University of North Carolina, Chapel Hill; 101 Centre for Ophthalmology and Visual Science, Lions Eye Institute, University of Western Australia, Crawley, Western Australia, Australia; 102 Department of Neonatology and Neonatal Intensive Care, The Children’s Memorial Health Institute, Al. Dzieci Polskich 20, Warsaw, Poland; 103 Princess Al-Jawhara Al-Brahim Centre of Excellence in Research of Hereditary Disorders (PACER-HD) King Abdulaziz University, Jeddah, Saudi Arabia; 104 Biomedical and Nutritional Sciences, University of Massachusetts, Lowell; 105 INSERM, UMR_S 1138, Centre de Recherche des Cordeliers, Paris, France; 106 University of Paris Diderot, Sorbonne Paris Cité, UMR_S 1138, Centre de Recherche des Cordeliers, Paris, France; 107 Sorbonne Universités, UPMC Univ Paris 06, UMR_S 1138, Centre de Recherche des Cordeliers, Paris, France; 108 Department of Internal Medicine, Hospital Clinic, IDIBAPS, Barcelona, Spain; 109 Department of Epidemiology and Population Genetics, Centro Nacional Investigación, Cardiovasculares (CNIC), Madrid, Spain; 110 College of Public Health, University of Kentucky, Lexington; 111 Department of Statistics and Applied Probability, Faculty of Science, National University of Singapore, Singapore; 112 Department of Public Health and Primary Care, Leiden University Medical Center, Leiden, Netherlands; 113 Friedman School of Nutrition Science & Policy, Tufts University, Boston, Massachusetts; 114 Department of Epidemiology, University of Washington, Seattle; 115 Department of Health Sciences, University of Washington, Seattle; 116 Kaiser Permanent Washington Health Research Institute, Seattle; 117 Carolina Center for Genome Sciences, University of North Carolina, Chapel Hill

## Abstract

**Importance:**

Observational studies have shown associations of birth weight with type 2 diabetes (T2D) and glycemic traits, but it remains unclear whether these associations represent causal associations.

**Objective:**

To test the association of birth weight with T2D and glycemic traits using a mendelian randomization analysis.

**Design, Setting, and Participants:**

This mendelian randomization study used a genetic risk score for birth weight that was constructed with 7 genome-wide significant single-nucleotide polymorphisms. The associations of this score with birth weight and T2D were tested in a mendelian randomization analysis using study-level data. The association of birth weight with T2D was tested using both study-level data (7 single-nucleotide polymorphisms were used as an instrumental variable) and summary-level data from the consortia (43 single-nucleotide polymorphisms were used as an instrumental variable). Data from 180 056 participants from 49 studies were included.

**Main Outcomes and Measures:**

Type 2 diabetes and glycemic traits.

**Results:**

This mendelian randomization analysis included 49 studies with 41 155 patients with T2D and 80 008 control participants from study-level data and 34 840 patients with T2D and 114 981 control participants from summary-level data. Study-level data showed that a 1-SD decrease in birth weight due to the genetic risk score was associated with higher risk of T2D among all participants (odds ratio [OR], 2.10; 95% CI, 1.69-2.61; *P* = 4.03 × 10^−5^), among European participants (OR, 1.96; 95% CI, 1.42-2.71; *P* = .04), and among East Asian participants (OR, 1.39; 95% CI, 1.18-1.62; *P* = .04). Similar results were observed from summary-level analyses. In addition, each 1-SD lower birth weight was associated with 0.189 SD higher fasting glucose concentration (β = 0.189; SE = 0.060; *P* = .002), but not with fasting insulin, 2-hour glucose, or hemoglobin A_1c_ concentration.

**Conclusions and Relevance:**

In this study, a genetic predisposition to lower birth weight was associated with increased risk of T2D and higher fasting glucose concentration, suggesting genetic effects on retarded fetal growth and increased diabetes risk that either are independent of each other or operate through alterations of integrated biological mechanisms.

## Key Points

**Question:**

Is birth weight associated with type 2 diabetes and glycemic traits?

**Findings:**

This mendelian randomization study found that a 1-SD decrease in birth weight due to the genetic risk score was associated with a higher risk of type 2 diabetes among European and East Asian populations. In addition, a 1-SD decrease in birth weight was associated with a 0.189-SD increase in fasting glucose concentration, but not with fasting insulin, 2-hour glucose, or hemoglobin A_1c_ level.

**Meaning:**

A genetic predisposition to lower birth weight was associated with an increased risk of type 2 diabetes and increased fasting glucose, suggesting potential mechanisms through which perturbation of the antenatal and early-life environment affect predisposition to diabetes in later life.

## Teaser

This mendelian randomization study examines the association of birth weight with type 2 diabetes and glycemic traits.

## Introduction

Type 2 diabetes (T2D) has become a worldwide epidemic, with more than 422 million patients in 2014.^1^ However, the etiology of T2D is not fully understood. Identifying potentially causal risk factors would help guide prevention of the disease.

The thrifty phenotype hypothesis postulates that fetal growth and nutrition play important roles in influencing susceptibility to T2D in later life.^2^ In observational studies, low birth weight, a widely used indicator for fetal growth restriction, has been consistently associated with higher risk of T2D^3,4^ and adverse glycemic traits^5^ in later life. However, both maternal socioeconomic status and unmeasured lifestyle factors might confound these associations; therefore, the causality of these observations remains to be determined. We hypothesized that birth weight may be causally associated with T2D risk and related traits such as fasting glucose concentration, insulin level, insulin resistance, and insulin sensitivity.

Mendelian randomization (MR) analysis has become widely used to assess the potential causal associations of environmental risk factors with disease.^6,7,8,9,10,11^ This method is analogous to a randomized clinical trial where randomization to genotype takes place at conception, and it is less likely to be affected by confounding and reverse causation.^7,12^ Previous analyses have provided compelling evidence that fetal genotype has substantial impact on early growth, as measured by birth weight.^13^

Therefore, in this study, we used the genetic variants for birth weight as an instrumental variable^14,15^ to perform an MR analysis to examine the association of birth weight with T2D and glycemic traits, using both study-level data and summary-level data.

## Methods

### Study Design

This study was conducted using summary association data generated by previous studies. Owing to the use of previously collected, deidentified, aggregated data, this study did not require institutional review board approval per the US Federal Policy for Protection of Human Research Subjects. Ethical approval was obtained for all original studies. Reporting of this study followed the Strengthening the Reporting of Observational Studies in Epidemiology (STROBE) reporting guideline.

Observational studies are prone to reverse causation, confounding, and biases and can generate unreliable findings in relation to the causal effects of modifiable exposures on disease outcomes. Mendelian randomization is a method aimed at unbiased detection of causal effects and estimation of their magnitudes (eMethods in the Supplement). To consistently estimate the causal effects, the genetic variants used in an MR analysis must satisfy 3 assumptions (eFigure 1 in the Supplement):^16^ (1) the genetic variants used as instrumental variables (IV) are associated with the exposure (birth weight); (2) the genetic variants are not associated with any confounder of the exposure-outcome association; and (3) the genetic variants are conditionally independent of the outcome (T2D and glycemic traits) given the exposure and confounders. The second and third assumptions are known as independence from pleiotropy.^16^

**Figure 1.**
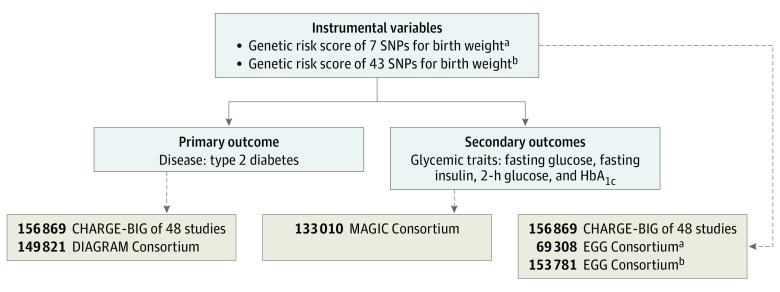
Study Design Sources of data for analysis included study-level data from the Cohorts for Heart and Aging Research in Genomic Epidemiology Birth Gene (CHARGE-BIG) Study (49 studies, n = 180 056 participants) and summary-level data from the Diabetes Genetics Replication and Meta-analysis (DIAGRAM) Consortium (n = 149 821 participants),^17^ the Meta-analyses of Glucose and Insulin-Related Traits (MAGIC) Consortium (n = 133 010 participants),^18,19,20,21,22^ and the Early Growth Genetics (EGG) Consortium (n = 153 781 participants).^13,23^ HbA_1c_ indicates hemoglobin A_1c_; and SNP, single-nucleotide polymorphism. ^a^Estimates of 7 SNPs for birth weight were extracted from the EGG Consortium (n = 69 308 participants).^23^ ^b^Estimates of 43 SNPs for birth weight were extracted from the EGG Consortium (n = 153 781 participants).^13^

The study design of this MR analysis consisted of 2 components^17,18,19,20,21,22,23^ (Figure 1). First, we explored the association of birth weight with risk of T2D using study-level data, including 49 cross-sectional and prospective cohort studies with a total of 180 056 participants, including 41 155 patients with T2D from the Cohorts for Heart and Aging Research in Genomic Epidemiology—Birth Gene Study (CHARGE-BIG). The primary IV was a genetic risk score (GRS) for birth weight using 7 single-nucleotide polymorphisms (SNPs) (*P* < 5 × 10^−8^) from a genome-wide association study (GWAS) in the Early Growth Genetics (EGG) Consortium.^23^ We analyzed the data within each study using standardized analytic methods. The IV estimator is calculated as the pooled β coefficient from the GRS-T2D association divided by the pooled β coefficient from the GRS–birth weight association. Second, we tested the association of birth weight with T2D and glycemic traits using summary-level data from the EGG Consortium (n = 153 781),^13,23^ the Diabetes Genetics Replication and Meta-analysis (DIAGRAM) Consortium (n = 149 821),^17^ and the Meta-analyses of Glucose and Insulin-Related Traits (MAGIC) Consortium (n = 133 010).^18,19,20,21,22^ In this study, the 7-SNP score^23^ was used as the main IV because the new GWAS that identified 60 SNPs for birth weight was published after the study-level results had already been run. Therefore, we used the 43 SNPs available in this analysis, a subset of the 60 SNPs,^13^ as the IV for birth weight in summary-level analyses.

### Study Populations and Data Sources

#### Study-Level Data

Study-level data including 49 cross-sectional and prospective cohort studies with up to 180 056 participants from the CHARGE-BIG were used (eTable 1 in the Supplement). Descriptions of each participating study are shown in the eAppendix in the Supplement. All participants provided written, informed consent, and ethical approval was granted by local ethics committees for participating studies (eTable 2 in the Supplement). Birth weight was collected by self-reported questionnaires or medical records in each study. Detailed information on the study-specific data collection methods is provided in eTable 2 in the Supplement. Covariates were measured using direct measurement or self-reported using questionnaire data from each study (eTable 2 in the Supplement). The primary outcomes were prevalence or incidence of T2D, defined based on report of T2D or current use of antidiabetes medication. Participants with missing values or those lost to follow-up were excluded. Precise information on the outcome for each study is reported in eTable 3 in the Supplement.

#### Selection of SNPs and GRS Calculation

Seven SNPs were identified as being associated with birth weight by a previous GWAS.^23^ All studies used direct genotype information on SNPs for birth weight from previously genotyped array data. Whenever a SNP was not genotyped directly, we used either (1) the HapMap II CEU (European) reference panel-imputed genetic information from GWAS or (2) genotype information from a predefined list of proxies that are in high linkage disequilibrium with the SNP (*r*^2^ > 0.8). Genotyping platforms, genotype frequencies, Hardy-Weinberg equilibrium *P* values, and call rates for the 7 SNPs are listed in eTable 4 and eTable 5 in the Supplement. To estimate the genetic predisposition to low birth weight, a GRS for low birth weight was calculated on the basis of these 7 well-established SNPs (eTable 6 in the Supplement).^23^ We assumed that each SNP in the panel acts independently in an additive manner, and the GRS was calculated using a weighted method (eAppendix in the Supplement).

#### Summary-Level Data

Summary-level data from the EGG Consortium,^13,23^ DIAGRAM Consortium,^17^ and MAGIC Consortium^18,19,20,21,22^ were used. For IV, both the 7-SNP GRS (explained between 0.32% and 1.52% of variance in birth weight) (eTable 6 and eTable 7 in the Supplement)^23^ and the 43-SNP GRS (explained 2.0% of variance in birth weight) (eTable 8 and eTable 9 in the Supplement)^13^ for birth weight were used from 2 previous GWAS studies in the EGG Consortium with up to 153 781 individuals. For T2D, data were obtained from the DIAGRAM Consortium; this study included 149 821 individuals of European descent.^17^ In addition to the primary outcomes of T2D, secondary outcomes of glycemic traits such as fasting glucose, fasting insulin, 2-hour glucose, and hemoglobin A_1c_ concentrations were examined (eTable 7 and eTable 9 in the Supplement). Data from the MAGIC Consortium with up to 133 010 individuals were used for glycemic traits. Informed consent was obtained from all participants of contributing studies. Contributing studies received ethical approval from their respective institutional review boards.

### Statistical Analysis

#### Study-Level Data

For study-level data from the CHARGE-BIG study, a standard analytic protocol was applied to each individual study to produce comparable results. Logistic regression was used to test the association of birth weight with risk of T2D after adjustment for age, sex, and other baseline covariates, where available (smoking status, physical activity, total energy intake, and alcohol intake). Linear regression was used to test the association of the GRS with birth weight after adjustment for age, sex, and principal components for population stratification (principal components analysis [PCA]). Logistic regression was used to test the association of the GRS with risk of T2D after adjustment for age, sex, and PCA. The inclusion of PCA as covariates is commonly used to correct for population stratification according to ancestral background.^24^

To validate assumption 1, that the GRS for birth weight was a strong IV for birth weight (eTable 10 in the Supplement), an *F* statistic for the IV was calculated in the Nurses’ Health Study (NHS) and Health Professionals Follow-up Study (HPFS) cohorts as a measure of the strength of IV for prediction of the birth weight, controlling for covariates (age, sex, PCA). An *F* statistic greater than 10 is evidence of a strong IV.^25^

To examine assumption 2, that GRS for birth weight was not associated with potential confounders, the association of the GRS with age, body mass index, smoking, alcohol use, and total energy intake was determined among individuals in the NHS and HPFS cohorts (eTable 11 in the Supplement).

Meta-analyses were conducted using study-level data from each study; we then pooled the β coefficients across studies, using random-effects or fixed-effects meta-analysis. Meta-analyses were conducted in Stata statistical software version 13.0 (StataCorp). All *P* values reported are 2-sided. We assessed heterogeneity with the *I*^2^ statistic. We assessed between-study heterogeneity via the Cochrane *Q* statistic and *I*^2^ statistics.^26,27,28^ For the proposed cutoff of *I*^2^ > 0.25, we found nonnegligible heterogeneity between studies, in particular among the birth weight–T2D associations, but also for the association between GRS and birth weight or T2D (*I*^2^ > 0.25). As a consequence, we used random-effects meta-analysis throughout. After meta-analysis, we used the IV estimators to quantify the strength of the association of birth weight with risk of T2D.^29^ The IV estimator, which is identical to that derived by the widely used 2-stage least-squares method,^30^ was calculated as the β of the regression coefficients for GRS-T2D and GRS–birth weight associations (eMethods in the Supplement).

#### Summary-Level Data

For the summary-level data from the EGG, DIAGRAM, and MAGIC consortia, the estimates of the association of birth weight with T2D risk and glycemic traits were pooled using the inverse-variance weighted, MR-Egger, and weighted-median methods for multiple genetic variants (eMethods in the Supplement). Detailed information on this MR method has been described previously.^31,32,33^

To examine assumption 3, that the IV for birth weight affects risk of T2D only through birth weight, but not through other pathways, the MR-Egger method was used (eMethods in the Supplement). Egger regression is a tool to detect small study bias in meta-analysis and it can be adapted to test for bias from type I pleiotropy, which is problematic for the interpretation of MR. Type I pleiotropy occurs when a single locus directly influences multiple phenotypes and is more pronounced at the level of the gene than at the level of single SNPs.^6^ Under the assumption that the association of each genetic variant with the exposure is independent of the pleiotropic effect of the variant (not via the exposure), the MR-Egger test gives a valid test of the null causal hypothesis.^16^ Using the MR-Egger method, the effect of the IV on the exposure is plotted against its effect on the outcome, and an intercept distinct from the origin provides evidence for pleiotropic effects. Additionally, the slope of the MR-Egger can provide pleiotropy-corrected causal estimates under a weaker assumption (the instrument strength independent of direct effect assumption).^16^

For analyses of both study-level data and summary-level data, the effect size for each meta-analysis is reported in the main results as the effect of a 1-SD change in birth weight or glycemic quantitative traits, as this metric is more interpretable than an arbitrary difference. Absolute risk increase (ARI) per 1000 participant-years for T2D was also calculated (eMethods in the Supplement). *P* < .05 was considered statistically significant. Analyses were performed using Stata statistical software version 13 (StataCorp) and R statistical software version 3.2.3 (R Project for Statistical Computing).

## Results

### Characteristics of the 49 Participating Studies

The characteristics of the 49 participating studies with up to 180 056 participants, including 41 155 patients with T2D, are presented in eTable 1 in the Supplement. Twenty-two studies reported the genetic association between GRS and birth weight, and 33 studies reported the genetic association between GRS and risk of T2D. A total of 41 155 patients with T2D and 80 008 control individuals without T2D provided study-level data. Data from the DIAGRAM Consortium included 34 840 patients with T2D and 114 981 control individuals, overwhelmingly of European descent.^17^ The MAGIC Consortium included 133 010 participants,^18,19,20,21,22^ and the EGG Consortium included 153 781 participants (Figure 1).^13,23^

### Results for Testing MR Assumptions

To validate MR assumptions 1 and 2, the NHS and HPFS cohorts were used to examine the associations of GRS with birth weight and potential confounders. We found that the GRS for birth weight was a strong IV (*F* > 18) (eTable 10 in the Supplement), thus validating assumption 1. In addition, no associations between the GRS and age, body mass index, smoking, alcohol use, and total energy intake were observed in the NHS and HPFS cohorts (eTable 11 in the Supplement), thus validating assumption 2.

### Association of Birth Weight With Risk of T2D

**Figure 2.**
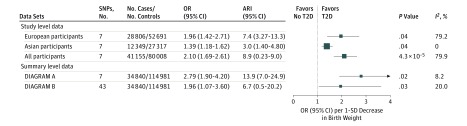
Mendelian Randomization of Birth Weight and Risk of Type 2 Diabetes (T2D) For type 2 diabetes, the data were analyzed from 49 studies from the Cohorts for Heart and Aging Research in Genomic Epidemiology Birth Gene Study where standardized analytic methods were used in individual study. This study included 41 155 patients with T2D and 80 008 controls. Data from the Diabetes Genetics Replication and Meta-analysis (DIAGRAM) Consortium included 34 840 patients with T2D and 114 981 controls, overwhelmingly of European descent. Summary results of 7 single-nucleotide polymorphisms (SNPs) for birth weight identified in genome-wide association studies were extracted from the Early Growth Genetics Consortium.^23^ Summary results for risk of T2D were extracted from the DIAGRAM Consortium.^17^ Summary results of 43 SNPs for birth weight were extracted from the Early Growth Genetics birth weight genome-wide association study.^13^ Summary results for risk of T2D were extracted from the DIAGRAM Consortium.^17^ We used the standard deviation value (543 g) from the birth weight genome-wide association study of the EGG Consortium.^13^ Results are standardized to a 1-SD lower birth weight owing to genetic risk score. ARI indicates absolute risk increase; OR, odds ratio

Study-level data showed that each 1-SD decrease in birth weight due to the GRS was associated with higher risk of T2D among all participants (odds ratio [OR], 2.10; 95% CI, 1.69-2.61; and ARI per 1000 participant-years, 8.9; 95% CI, 0.2-9.0; *P* = 4.03 × 10^−5^), among European participants (OR, 1.96; 95% CI, 1.42-2.71; and ARI per 1000 participant-years, 7.48; 95% CI, 3.27-13.34; *P* = .04)^34^ (Table 1), and among East Asian participants (OR, 1.39; 95% CI, 1.18-1.62; and ARI per 1000 participant-years, 3.04; 95% CI, 1.40-4.84; *P* = .04) (Figure 2; eFigure 2 and eFigure 3 in the Supplement). We did not find a significant difference in OR for T2D between MR estimates and conventional observational results (OR, 1.41 per 1-SD lower birth weight; 95% CI, 1.16-1.66) from 11 studies of the CHARGE-BIG (*P* = .86) (eFigure 4 in the Supplement).

**Table 1.** Mendelian Randomization of Birth Weight and Risk of Type 2 Diabetes

MR Estimates^a^	Summary Data A^b^		Summary Data B^b^	
	OR (95% CI)	*P* Value	OR (95% CI)	*P* Value
Simple median–based method^c^	1.57(1.24 to 2.00)	2.0 × 10-4	1.24(1.09 to 1.41)	.001
Weighted median–based method^c^	1.52(1.24 to 1.86)	1.1 × 10-4	1.29(1.13 to 1.47)	6.0 × 10-4
Inverse-variance–weighted method^c^	1.69(1.12 to 2.55)	.045	1.36(1.14 to 1.62)	.001
MR-Egger method^c^	2.79(1.90 to 4.20)	.02	1.96(1.07 to 3.60)	.03
MR-Egger regression^d^	0.007 (−0.081 to 0.095)	.94	0.011 (−0.002 to 0.02)	.22

Abbreviations: MR, mendelian randomization; OR, odds ratio.

^a^ In an MR framework, genetic variants for birth weight were assumed to influence type 2 diabetes only through birth weight, not through other pathways. In the present study, we used MR-Egger regression to assess for the presence of pleiotropy.^16^ This approach is based on Egger regression, which was used to assess publication bias in the meta-analysis.^34^ Using the MR-Egger method, the β coefficient of the MR-Egger regression provides pleiotropy-corrected causal estimates and an intercept distinct from the origin provides evidence for pleiotropic effects.^16^

^b^ Sample sizes of patients with type 2 diabetes and control individuals were 12 171 and 56 862 for both summary data A and summary data B. Number of single-nucleotide polymorphisms used of summary data A and summary data B are 7 and 43, respectively. Number of participants with birth weight in summary data A and summary data B are 69 308 and 153 781, respectively.

^c^ We used simple median–based method, weighted median–based method, inverse-variance–weighted method, and MR-Egger method to provide consistent results for causal effect of birth weight on type 2 diabetes.

^d^ Values in this row are intercept (95% CI).

We further conducted stratified analyses of estimated causality by age, sex, body mass index, ethnic group, sample size, study design, and number of SNPs included. An association of birth weight with T2D was observed among both men and women, both obese and normal-weight participants, and both European and East Asian participants. However, evidence for a causal association was not observed in the subsample of individuals younger than 50 years (Table 2).

**Table 2.** Stratified Analyses of Estimated Causality Between Birth Weight and Risk of Type 2 Diabetes

Subgroup	Genetic Association of Birth Weight per SD^a^			Genetic Association of Type 2 Diabetes			Estimated Causality^b^	
	No. of Studies	β (95% CI)	*P* Value	No. of Studies	β (95% CI)	*P* Value	OR (95% CI)	*P* Value
Age, y	23							
≥50		0.04 (0.03 to 0.05)	3.6 × 10^−4^	28	0.03 (0.01 to 0.05)	.0004	2.12 (1.70 to 2.64)	.0006
<50				5	0.04 (−0.10 to 0.02)	.18	1.67 (0.87 to 5.65)	.18
Sex								
Male	17	0.04 (0.02 to 0.05)	8.4 × 10^−4^	24	0.03 (0.01 to 0.05)	.006	1.89 (1.46 to 2.46)	.02
Female	16	0.04 (0.01 to 0.06)	9.4 × 10^−4^	23	0.03 (0.01 to 0.04)	.002	2.10 (1.49 to 2.97)	.03
Body mass index^c^	23							
≥25		0.04 (0.03 to 0.05)	3.6 × 10^−4^	25	0.02 (0.00 to 0.04)	.02	1.81 (1.39 to 2.37)	.03
<25				8	0.04 (0.02 to 0.06)	<.001	2.82 (2.20 to 3.60)	3.1 × 10^−5^
Ethnic group								
European	22	0.04 (0.03 to 0.05)	3.6 × 10^−4^	24	0.03 (0.01 to 0.05)	.02	1.96 (1.42 to 2.71)	.04
East Asian	1	0.09 (0.00 to 0.18)	5.1 × 10^−3^	9	0.03 (0.02 to 0.04)	<.001	1.39 (1.18 to 1.62)	.04
Sample size, No.	23							
≥1500		0.04 (0.03 to 0.05)	3.6 × 10^−4^	27	0.03 (0.01 to 0.04)	.001	1.96 (1.58 to 2.44)	.002
<1500				6	0.07 (0.03 to 0.12)	<.001	3.45 (2.41 to 6.19)	.003
Study design	23							
Cohort		0.04 (0.03 to 0.05)	3.6 × 10^−4^	26	0.03 (0.01 to 0.05)	<.001	2.06 (1.64 to 2.60)	.002
Case-control				5	0.02 (−0.03 to 0.06)	.47	1.55 (0.85 to 2.84)	.47
Cross-sectional				2	0.06 (−0.01 to 0.16)	.19	3.26 (0.89 to 7.02)	.19
No. of single-nucleotide polymorphisms	23							
7		0.04 (0.03 to 0.05)	3.6 × 10^−4^	27	0.03 (0.01 to 0.05)	.003	2.17 (1.65 to 2.87)	.005
<7				6	0.03 (0.01 to 0.04)	.0004	1.91 (1.58 to 2.31)	.0007

Abbreviation: OR, odds ratio.

^a^ Results were standardized to a 1-SD decrease in birth weight due to genetic risk score. The standard deviation was 543 g from the Early Growth Genetics Consortium.^13^

^b^ The estimates were derived from 49 studies from the Cohorts for Heart and Aging Research in Genomic Epidemiology Birth Gene Study where standardized analytic methods adjusted for confounders such as age, body mass index, sex, and the first 3 principal components for population stratification were used in individual study. In a mendelian randomization framework, the association between genetic risk score and type 2 diabetes is assumed to be independent of confounding factors. In our study, the instrumental variable estimator is calculated as the β coefficient from the association of genetic risk score with type 2 diabetes divided by the β coefficient from the association of genetic risk score with birth weight. These results are supportive of a causal, nonconfounded association.

^c^ Calculated as weight in kilograms divided by height in meters squared.

Summary-level data showed a similar association of low birth weight with risk of T2D when using the 7 SNPs (OR, 2.79; 95% CI, 1.90-4.20; and ARI per 1000 participant-years, 13.96; 95% CI, 7.02-24.96; *P* = .02) and when using 43 SNPs (OR, 1.86; 95% CI, 1.07-3.60; and ARI per 1000 participant-years, 6.70; 95% CI, 0.55-20.28; *P* = .03) (Figure 2). We further excluded previously reported loci for T2D such as *CDKAL1, ADCY5, BCAR1, HHEX/IDE, GCK, MTNR1B,* and *ANK1,* and low birth weight remained associated with risk of T2D (OR, 1.75; 95% CI, 1.05-3.16; *P* = .04).

### Association of Birth Weight With Glycemic Quantitative Traits

Using the weighted median–based method, we found that a 1-SD lower birth weight due to the GRS was associated with 0.189 SD higher fasting glucose concentration (β = 0.189; SE = 0.060; *P* = .002) at the Bonferroni-adjusted level of significance (*P* < .01). Consistently, the inverse-variance–weighted analysis also showed an association of birth weight with fasting glucose concentration (0.207 SD higher fasting glucose concentration per 1-SD lower birth weight; β = 0.0.207; SE = 0.073; *P* = .03) (Table 3). These findings were replicated using the 43 SNPs as an IV, suggesting robustness of our findings. However, there was no evidence for an association of birth weight with other glycemic traits such as fasting insulin, 2-hour glucose, or hemoglobin A_1c_ concentrations (Table 3).

**Table 3.** Mendelian Randomization Analyses of Birth Weight and Glycemic Quantitative Traits^a^

Data Source	SD	No.		MR Estimates, Units of SD per 1-SD Decrease in Birth Weight						MR-Egger Regression	
				Weighted Median–Based Method		Inverse-Variance–Weighted Method		MR-Egger Method			
		SNPs	Participants	β (SE)	*P* Value	β (SE)	*P* Value	β (SE)	*P* Value	Intercept (SE)	*P* Value
Fasting glucose, mg/dL											
Summary data A^b^	13.1	7	133 010	0.189 (0.060)	.002	0.207 (0.073)	.03	0.113 (0.341)	.74	0.005 (0.017)	.78
Summary data B^c^	13.1	43	133 010	0.109 (0.049)	.03	0.415 (0.105)	.04	0.031 (0.099)	.23	−0.018 (0.010)	.07
Fasting insulin, log (pmol/L)											
Summary data A^b^	0.44	7	108 557	0.089 (0.096)	.36	0.021 (0.108)	.86	0.131 (0.502)	.79	−0.006 (0.026)	.82
Summary data B^c^	0.44	43	108 557	0.033 (0.082)	.69	0.050 (0.060)	.41	−0.027 (0.213)	.90	0.002 (0.006)	.70
2-h glucose, mg/dL^d^											
Summary data A^b^	10.1	7	42 854	0.494 (0.352)	.16	0.563 (0.411)	.22	−0.584 (1.851)	.75	0.060 (0.094)	.52
Summary data B^c^	10.1	43	42 854	0.406 (0.254)	.11	0.319 (0.203)	.12	0.378 (0.727)	.60	−0.002 (0.022)	.93
Hemoglobin A_1c_, % of total hemoglobin											
Summary data A^b^	0.54	7	46 368	0.118 (0.072)	.10	0.186 (0.084)	.07	0.135 (0.390)	.73	0.003 (0.020)	.89
Summary data B^c^	0.54	43	46 368	0.038 (0.063)	.55	0.086 (0.069)	.22	0.158 (0.242)	.51	−0.002 (0.007)	.76

Abbreviations: HbA_1c_, hemoglobinA_1c_; MR, mendelian randomization; SNP, single-nucleotide polymorphism.

SI conversion factor: To convert glucose to mmol/L, multiply by 0.0555; HbA_1c_ to proportion of total hemoglobin, multiply by 0.01.

^a^ Results were standardized to a 1-SD decrease in birth weight due to genetic variants. For birth weight, 1-SD was assumed to correspond to 543 g, the pooled results from the Early Growth Genetics (EGG) Consortium.^23^ The Meta-analyses of Glucose and Insulin-Related Traits (MAGIC) Consortium did not report estimates of variants in units of standard deviations. β values from this consortium were standardized so that the association of birth weight with glycemic traits could be uniformly expressed in terms of standard deviations. For fasting glucose, 2-hour glucose, and HbA_1c_ from the MAGIC Consortium, 1 SD was assumed to correspond to 13.1 mg/dL, 10.1 mg/dL, and 0.535%, respectively, the pooled SD of studies included in a previous report from the MAGIC Consortium.^18^ The threshold of significance was at the Bonferroni-adjusted level *P* < .01 (0.05 / 4 = 0.01).

^b^ Estimates of 7 SNPs for birth weight were extracted from EGG Consortium.^23^ For glycemic traits, estimates were derived from the MAGIC Consortium (n = 133 010 participants).^18,19,20,21,22^

^c^ Estimates of 43 SNPs for birth weight were extracted from EGG Consortium.^13^ For glycemic traits, estimates were derived from the MAGIC Consortium (n = 133 010 participants).^18,19,20,21,22^

^d^ Two-hour glucose refers to measured blood glucose concentration 2 hours after consumption of dissolved glucose.

### Sensitivity Analyses of MR

In sensitivity analyses, we used 4 different methods (simple median based, weighted median based, inverse-variance weighted, and MR-Egger) to estimate the association of birth weight with risk of T2D using summary-level data. The results showed consistent associations (Table 2), indicating robustness of our findings. We further conducted a sensitivity analysis of association of birth weight with risk of T2D using 8 studies providing both GRS–birth weight and GRS-T2D associations (eFigure 5 in the Supplement) in the CHARGE-BIG study. Similarly, we found that each 1-SD lower birth weight due to the GRS was associated with higher risk of T2D (OR, 2.66; 95% CI, 1.30-4.02; *P* = 6.76 × 10^−4^), providing further evidence of finding robustness.

To examine MR assumption 3, we further tested whether any of the selected SNPs were influenced by linkage disequilibrium and pleiotropy. We found that none of the SNPs were in linkage disequilibrium with each other (*r*^2^ > 0.05). In addition, the intercept term estimated from MR-Egger was centered at the origin with a confidence interval including the null (0.007; 95% CI −0.081 to 0.095; *P* = .94) (Table 1), suggesting the results were not influenced by pleiotropy. For glycemic traits, the intercept (SE) from MR-Egger regression also suggested that the observed results were not influenced by pleiotropy (Table 3).

## Discussion

In the largest MR study thus far, to our knowledge, we investigated a potential causal role of birth weight in the development of T2D and regulation of glycemic traits using study-level data and summary-level data. Our results show that genetically determined lower birth weight was associated with increased risk of T2D and elevated fasting glucose concentration, supporting an association between lower birth weight and development of T2D.

Compelling observational studies have shown that lower birth weight is associated with a higher T2D risk.^3,4,35,36,37,38,39,40^ For example, data from a meta-analysis of 30 studies found an inverse birth weight–T2D association; the pooled OR of T2D was 1.13 (95% CI, 1.10-0.1.17) per kilogram decrease in birth weight.^3^ However, in most of the observational studies included in this meta-analysis, birth weight was associated with potential confounders. Therefore, residual confounding may have contributed to the observed associations, illustrating a major limitation of observational studies in inference of causality. In the present study, we used MR analysis to minimize the potential confounding effect. The GRS used in our study was not correlated with potential confounders, and was validated as a strong and reliable IV for birth weight.^15^ Therefore, our findings concur with a previous study^15^ and lend genetic support to prior evidence of observational association between birth weight and risk of T2D.

Our findings suggest that birth weight may be a useful target for a prevention strategy to mitigate T2D risk in later life. According to the thrifty phenotype hypothesis,^2^ the observed associations may originate in utero where intrauterine growth restriction affects epigenetic alterations and alters intracellular insulin-signaling pathways.^41,42^ Such permanent alterations in structure, physiology, and metabolism are thought to result in key disruptions to the endocrine system.^42,43^ It has been suggested that the public health implications of the inverse birth weight–T2D association depend on the precise nature of the underlying causal exposure and its amenability to change.^3^ Our MR results demonstrate that birth weight itself is a causal exposure, implying the public health impact of birth weight modification. Interestingly, previous interventions for increasing birth weight through changes in maternal nutrition have increased birth weight by up to 200 g in populations.^44^ Such an increase in birth weight could translate into a reduction in T2D risk of up to 10%.^3^ Therefore, our findings highlight the potential importance of improving fetal growth and nutrition in the prevention of T2D. In addition, ongoing research to understand the mechanistic links between the genetic loci that influence birth weight may lead to novel therapeutic strategies to modify birth weight and subsequently reduce the risk of T2D.^45^ Importantly, our findings are of public health significance and may help in understanding the mechanisms by which low birth weight increases risk of T2D.

The MR analysis used in this study satisfied 3 assumptions. Assumption 1 requires a strong link between the genetic variants used as an IV and birth weight. The GRS used in our study was demonstrated to be a strong IV with an *F* statistic greater than 18.^31^ For assumption 2, MR assumes the IV (GRS) was not associated with potential confounders. Study-level results showed that GRS was not associated with potential confounders; nevertheless, we could not exclude the possibility that our results might be affected by unmeasured confounders. For assumption 3, MR assumes that the IV for birth weight affects risk of T2D only through birth weight, but not through other pathways. To validate assumption 3,^16^ the intercept term estimated from MR-Egger regression was centered at the origin with a confidence interval including the null, suggesting that our results were not influenced by pleiotropy.

Our current study has several other strengths. First, to our knowledge, our study is the largest MR analysis assessing the association of birth weight with T2D risk and glycemic traits to date. The large sample size allowed us to assess the consistency of associations across studies and to gain sufficient power for conclusive estimation of associations. Second, sensitivity analyses of 2 different data sources (study-level and summary-level data sets) were conducted. The steps taken in this study reduced the risk of bias and pleiotropy. Importantly, the consistent associations estimated from complementary MR approaches, such as the weighted median regression method, inverse-variance–weighted method, and MR-Egger method, support the robustness of our findings. Finally, most of the studies included were homogeneous, and we used standardized methods and performed the analysis individually in each study. Therefore, the effect of population stratification on the instrumental results should be minimal.

### Limitations

This study has some limitations, and the results should be interpreted with sufficient caution. Although the MR method is theoretically well established, we recognize that there are still many limitations in practice. First, we assumed that the associations of birth weight with T2D and glycemic traits were linear. Indeed, several observational studies suggested U-shaped associations.^46,47,48,49^ Therefore, further investigations employing a nonlinear MR approach are warranted to investigate the causality. In addition, we only used 7 SNPs in study-level analyses; this may lead to a weak IV, and thus introduce bias. Second, although the MR-Egger method suggested that our results were not affected by pleiotropy, it is possible that the shared genetic basis between birth weight and T2D may also contribute to the association. Third, although previous evidence indicated that variation in the fetal genome was the predominant driver of the birth weight associations,^13^ birth weight may be influenced by both fetal and correlated maternal genotypes. Given the correlation (*r* of approximately 0.5) between maternal and fetal genotype,^13^ we could not exclude the possibility that associations between fetal genotype and birth weight may result from indirect effects of the maternal genotype influencing birth weight via the intrauterine environment.^13^ In addition, recent GWAS identified several novel loci for offspring birth weight and highlighted maternal genetic effects that are independent of fetal genetics.^50^ Therefore, there are limitations in assuming causality on the basis of fetal genotype and fetal phenotype associations; dissecting maternal and fetal effects on adult T2D risk are also needed. In addition, maternal genetic variants could influence both offspring birth weight and other aspects of nurturing. Cross-generation MR studies are susceptible to such dynamic effects, which introduce potential biases. Therefore, future cross-generation MR studies should consider ways to reduce the biases that might result from these assumption violations.^51^

Fourth, the widely used GRS may not fit the assumptions for an IV well. Furthermore, most of the risk factors are not static, but dynamic, and this may only be captured by taking both genetic and environmental factors into account over time. Fifth, assumption 3, that genetic markers affect T2D only through birth weight, is sometimes referred to as the exclusion restriction. The MR assumptions are violated if the genetic marker affects T2D through pathways other than through birth weight, which may lead to substantial biases in MR analysis.^52^ We cannot exclude the possibility that genetic markers for birth weight were associated with other pathways that influence T2D risk. In the present study, we used only the MR-Egger method to examine assumption 3. Although we found that the results were not influenced by pleiotropy, the MR-Egger analysis has limitations and is not able to reliably detect a dose-response relationship in the genetic associations with birth weight and with T2D, and hence cannot distinguish between pleiotropy and causal effect.^53^ Sixth, even though many relevant covariates, including age, sex, ethnicity, region, total energy, and PCA were included in the statistical models, residual and unmeasured confounding cannot be ruled out. Many established confounders, such as maternal diet, lifestyle, and additional genetic markers, may confound the association of birth weight with T2D risk. Further investigation considering these confounders is needed. In addition, given that the studies involved are overwhelmingly in non-Hispanic white populations, testing for generalizability to other ethnic groups warrants further investigation.

## Conclusions

This study found that genetic predisposition to lower birth weight was associated with increased risk of T2D and impaired fasting glucose concentration. Our results suggest the presence of genetic effects on retarded fetal growth and increased diabetes risk that either are independent of each other or operate through alterations of integrated biological mechanisms.

## Notes

Supplement. **eMethods.** Mendelian Randomization Method **eFigure 1.** Schematic Representation of a Mendelian Randomization Approach **eFigure 2.** Genetic Association With Birth Weight **eFigure 3.** Genetic Association With Risk of T2DM **eFigure 4.** Association of Birth Weight With Risk of T2DM **eFigure 5.** Causality Estimated From Individual Study **eTable 1.** Baseline Characteristics of Included 49 Studies in the CHARGE-BIG Study **eTable 2.** Assessment of Birth Weight and Covariates in the CHARGE-BIG Study **eTable 3.** Assessment of Type 2 Diabetes in the CHARGE-BIG Study **eTable 4.** Genotyping Information in the CHARGE-BIG Study **eTable 5.** Distribution of Genotypes of Included 7 SNPs in the CHARGE-BIG Study **eTable 6.** Associations Between Seven Loci Associated With Birth Weight and Various Anthropometric Measures Taken at Birth (Data From Summary Results) **eTable 7.** Genetic Association of Birth Weight Genetic Variants With Glycemic Traits (Data From Summary Results) **eTable 8.** Sixty Loci Associated With Birth Weight (*P*<5×10^−8^) in European Ancestry and/or Trans-Ancestry (Data From Summary Results) **eTable 9.** Genetic Association of Birth Weight Related 60 Genetic Variants With Glycemic Traits (Data From Summary Results) **eTable 10.** Association of the Genetic Risk Score With Birth Weight and F Statistic for the Instrumental Variable in the NHS, HPFS, and WHI Cohorts **eTable 11.** Association of Birth Weight Genetic Risk Score With Confounders According to Quartiles of the GRS in the NHS, HPFS and WHI Studies **eAppendix.** Description of Included Studies **eReferences**
