# Supplementary material for: Association of Birth Weight With Type 2 Diabetes and Glycemic Traits: A Mendelian Randomization Study
Source: JAMA Netw Open. 2019 Sep 20;2(9):e1910915. doi: 10.1001/jamanetworkopen.2019.10915 (PMC6755534; doi:10.1001/jamanetworkopen.2019.10915)
Supplement: Supplement. — eMethods. Mendelian Randomization Method eFigure 1. Schematic Representation of a Mendelian Randomization Approach eFigure 2. Genetic Association With Birth Weight eFigure 3. Genetic Association With Risk of T2DM eFigure 4. Association of Birth Weight With Risk of T2DM eFigure 5. Causality Estimated From Individual Study eTable 1. Baseline Characteristics of Included 49 Studies in the CHARGE-BIG Study eTable 2. Assessment of Birth Weight and Covariates in the CHARGE-BIG Study eTable 3. Assessment of Type 2 Diabetes in the CHARGE-BIG Study eTable 4. Genotyping Information in the CHARGE-BIG Study eTable 5. Distribution of Genotypes of Included 7 SNPs in the CHARGE-BIG Study eTable 6. Associations Between Seven Loci Associated With Birth Weight and Various Anthropometric Measures Taken at Birth (Data From Summary Results) eTable 7. Genetic Association of Birth Weight Genetic Variants With Glycemic Traits (Data From Summary Results) eTable 8. Sixty Loci Associated With Birth Weight (P<5×10^−8^) in European Ancestry and/or Trans-Ancestry (Data From Summary Results) eTable 9. Genetic Association of Birth Weight Related 60 Genetic Variants With Glycemic Traits (Data From Summary Results) eTable 10. Association of the Genetic Risk Score With Birth Weight and F Statistic for the Instrumental Variable in the NHS, HPFS, and WHI Cohorts eTable 11. Association of Birth Weight Genetic Risk Score With Confounders According to Quartiles of the GRS in the NHS, HPFS and WHI Studies eAppendix. Description of Included Studies eReferences [file jamanetwopen-2-e1910915-s001.pdf]

Supplementary Online Content

BIRTH-GENE (BIG) Study Working Group. Association of birth weight with type 2 diabetes and glycemic traits: a mendelian randomization study. *JAMA Netw Open*. 2019;2(9):e1910915. doi:10.1001/jamanetworkopen.2019.10915

eMethods. Mendelian Randomization Method

eFigure 1. Schematic Representation of a Mendelian Randomization Approach

eFigure 2. Genetic Association With Birth Weight

eFigure 3. Genetic Association With Risk of T2DM

eFigure 4. Association of Birth Weight With Risk of T2DM

eFigure 5. Causality Estimated From Individual Study

eTable 1. Baseline Characteristics of Included 49 Studies in the CHARGE-BIG Study

eTable 2. Assessment of Birth Weight and Covariates in the CHARGE-BIG Study

eTable 3. Assessment of Type 2 Diabetes in the CHARGE-BIG Study

eTable 4. Genotyping Information in the CHARGE-BIG Study

eTable 5. Distribution of Genotypes of Included 7 SNPs in the CHARGE-BIG Study

eTable 6. Associations Between Seven Loci Associated With Birth Weight and Various Anthropometric Measures Taken at Birth (Data From Summary Results)

eTable 7. Genetic Association of Birth Weight Genetic Variants With Glycemic Traits (Data From Summary Results)

eTable 8. Sixty Loci Associated With Birth Weight ($P<5\times10^{-8}$) in European Ancestry and/or Trans-Ancestry (Data From Summary Results)

eTable 9. Genetic Association of Birth Weight Related 60 Genetic Variants With Glycemic Traits (Data From Summary Results)

eTable 10. Association of the Genetic Risk Score With Birth Weight and *F* Statistic for the Instrumental Variable in the NHS, HPFS, and WHI Cohorts

eTable 11. Association of Birth Weight Genetic Risk Score With Confounders According to Quartiles of the GRS in the NHS, HPFS and WHI Studies

eAppendix. Description of Included Studies

eReferences

This supplementary material has been provided by the authors to give readers additional information about their work.

© 2019 BIRTH-GENE (BIG) Study Working Group. *JAMA Network Open.*

eMethods: Mendelian Randomization Method

Study-level data

Birth weight SNP selection and data sources

To explore the causal effect of birth weight (BW) on T2DM (T2DM) in 49 individual studies from the CHARGE-BIG study, we calculated a genetic risk score using 7 SNPs for BW identified from previous EGG consortium. Genetic variants associated with BW at a genome-wide significant level ($P < 5 \times 10^{-8}$) were obtained from EGG consortium with up to 153,781 individuals.[1]

Genetic risk score calculation

To estimate the genetic predisposition to low BW, a genetic risk score (GRS) was calculated on the basis of the 7 above-mentioned SNPs (**eTable 1**).[1] We assumed that each SNP in the panel acts independently in an additive manner, and the genetic score was calculated by using a weighted method. Each SNP was weighted by its relative effect size (β-coefficient) obtained from the reported meta-analysis data.[1] We firstly created a weighted score using the equation: weighted score = $\beta_1 \times SNP_1 + \beta_2 \times SNP_2 + \ldots + \beta_n \times SNP_n$, where β is the β-coefficient for each individual SNP, and n is number of SNPs. To reflect the number of BW-decreasing allele, we rescaled the weighted score using the following equation: weighted genetic risk score = weighted score × (total number of SNPs / sum of the β-coefficients).

Meta-analysis and between-study heterogeneity

We assessed between-cohort heterogeneity via Cochrane's Q-statistic and I^2-statistics.[2-4] Histograms of the distribution of I^2 were shown in the associations of BW with T2DM and glycemic traits, as well as the associations of the BW related GRS with glycemic traits. For the proposed cut-off of $I^2>0.25$, it means there is non-negligible heterogeneity between studies for associations. If there is non-negligible heterogeneity, we used random-effects meta-analysis throughout, otherwise, fixed effects meta-analysis were used. [5]

Standard errors and inference for the instrumental variable (IV) estimator

After meta-analysis, we used the IV estimators to quantify the strength of the causal association of BW and T2DM.[6] The IV estimator, which is identical to that derived by the widely used two-stage least squares method,[7] was calculated as the β of the regression coefficients of GRS-T2DM and GRS-BW:

$$\beta_{IV} = \frac{\beta_{GRS\_T2D}}{\beta_{GRS\_BW}}$$

The standard error was calculated via the delta method as

$$se_{IV} = abs($$

Based on these estimates, we appealed to standard-normal asymptotics, with the resulting Wald test statistic and 95% confidence intervals given as

$$t_{IV} = \frac{\beta_{IV}}{se_{IV}} \qquad CI_{IV} = \beta_{IV} \pm 1.96\, se_{IV}$$

The p-value for the H_0: $\beta_{IV} = 0$ was derived from the standard normal distribution. For the association with T2DM, the 95% CI estimates were back-transformed through the antilog and exponentiation, respectively.

For comparing the IV estimate β_{IV} and the conventional estimate β_{BW_T2DM}, we considered the difference

© 2019 BIRTH-GENE (BIG) Study Working Group. *JAMA Network Open.*

$$\beta_{Diff} = \beta_{IV} - \beta_{BW\_T2D}$$

The corresponding standard error is

$$se_{Diff} = \sqrt{(}$$

We used again standard normal asymptotics for the difference, namely,

$$t_{Diff} = \frac{\beta_{Diff}}{se_{Diff}} \qquad CI_{Diff} = \beta_{Diff} \pm 1.96\, se_{Diff}$$

The p-value for the H_0: $\beta_{Diff} = 0$ was derived from the standard normal distribution, and the confidence intervals were back-transformed as above.

Based on IV estimates, we tested the null hypothesis of no difference between the respective IV estimator and the conventional regression-based estimator of the effect of BW on T2DM by comparing the IV estimate β_{IV} and the conventional estimate β_{BW_T2DM}, and then we used standard normal asymptotics for the difference, namely, $t_{Diff=}\beta_{Diff}$ / se_{Diff}. The p-value for the null hypothesis was derived from the standard normal distribution.

Summary-level data

BW SNP selection and data sources

Both 7-SNP GRS[1] and 60-SNP GRS[8] for BW were calculated based on SNPs identified from previous two GWAS studies by EGG consortium, respectively.[1,8] To obtain precise estimates of the genetic association of BW GRS with T2DM and glycemic traits, summary data from the DIAGRAM consortium including 149,821 individuals of European descent for T2DM,[9] and summary data from MAGIC consortium with up to 133,010 individuals for glycemic traits were used, respectively.

Linkage disequilibrium assessment

One requirement of MR analysis is that the selected SNPs must not be in linkage disequilibrium (LD) since if a selected SNP is highly correlated with other risk loci, this may result in confounding.[10] In order to verify that the SNPs in this study met this requirement, we measured LD between all selected SNPs using CEU samples from the 1000 Genomes Project using SNAP (https://archive.broadinstitute.org/mpg/snap/ldsearchpw.php).

MR estimates

For the summary-level data from EGG, DIAGRAM, and MAGIC consortium, we conducted our MR analyses using summarized data, in particular the associations (beta-coefficients and standard errors) of each genetic variant with the BW and T2DM risk and glycemic traits.[11] The estimates of the causal effect of BW on T2DM risk and glycemic traits were pooled by using the inverse-variance weighted method,[12] MR-Egger method [13] and weighted median method.[14] Detailed information on these MR methods have been described previously.[12,15] It was recommended to use all these methods when there are multiple genetic variants to assess the robustness of any causal finding to different sets of assumptions.[16] The proposed inverse-variance weighted method gives the same estimates using these summarized data as the well-established two-stage least squares method that uses individual-level data.[12] The inverse-variance weighted (IVW) method provides a consistent estimate of the causal effect of BW on T2DM risk and glycemic traits when each of the genetic variants satisfies the assumptions of an instrumental variable.[12] Two further methods such as the MR-Egger method[13] and the weighted median method[14] have been proposed for providing consistent causal estimates from summarized data for multiple genetic variants.

Pleiotropy assessment and sensitivity analyses

© 2019 BIRTH-GENE (BIG) Study Working Group. *JAMA Network Open.*

MR analyses assume that the chosen SNPs do not exert pleiotropic effects on the outcomes by operating through biological pathways independent of the exposure. However, in MR analyses, a SNP may influence the outcome via other factors if the SNP acts upon the other factors through the exposure itself.[10] The inclusion of SNPs that contribute through a pleiotropic pathway could bias estimates. This can be difficult to test when examining BW, which is influenced by many different environmental factors and physiological mechanisms. In this study, we used MR-Egger regression to detect the presence of pleiotropy.[13] In brief, the approach is based on Egger regression, which has been used to examine publication bias in the meta-analysis literatures. Using the MR-Egger method, the SNP's effect upon the exposure variable is plotted against its effect upon the outcome, and an intercept distinct from the origin provides evidence for pleiotropic effects. Additionally, the slope of the MR-Egger regression can provide pleiotropy-corrected causal estimates. An important condition of this approach is that the SNP-exposure association must be independent of the SNP's direct effects upon the outcome, which may not always be satisfied in cases where all pleiotropic effects are attributed to a single confounder. Nonetheless, the MR-Egger method can provide unbiased estimates even if all the chosen SNPs are invalid.[13]

In addition, the weighted median approach was used to examine causal effect and pleiotropy.[14] Using this method, MR estimates are ordered and weighted by the inverse of their variance. The weighted median approach offers some important advantages over MR-Egger because it improves precision. Therefore, the inverse-variance weighted, MR-Egger and weighted median methods were considered as sensitivity analyses for MR investigations with multiple genetic variants.[12,14,15]

For analyses of both study-level data and summary-level data, the effect size for each meta-analysis was reported in the main results as the effect on a one-standard-deviation (1-SD) change in BW or glycemic quantitative traits, because this metric is more interpretable than an arbitrary difference. Analyses were performed using Stata version 12 (StataCorp) and R version 3.2.3 (R Project for Statistical Computing). The threshold of statistical significance for T2DM as the primary outcome was $P < 0.05$. The threshold of significance for the analysis of glycemic traits as secondary outcomes was $P < 0.01$ ($0.05/4 = 0.01$).

Standardization of MR Estimates

Data (β values) from the CHARGE-BIG study were standardized so that the association of BW with T2DM risk could be uniformly expressed in terms of standard deviations. For BW, 1-SD was assumed to correspond to 543 gram, according to the pooled SD from the EGG consortium.[1]

The EGG consortium reported estimates of variants in units of standard deviations of BW, while the MAGIC consortium did not. Therefore, β values from the MAGIC consortium were also standardized so that the association of BW with T2DM risk and glycemic traits could be also uniformly expressed in terms of standard deviations.

For fasting glucose, two-hour glucose and HbA1c from the MAGIC consortium, 1-SD was assumed to correspond to 13.1 mg/dl, 10.1 mg/dl and 0.535%, respectively, according to the pooled SD of studies included in a previous report from the MAGIC consortium.[17] 1-SD of 0.44 for log-transformed fasting insulin from Europeans was used.[18]

Calculation of absolute risk increases

To estimate the absolute risk increase based on calculated odds ratio estimated for T2DM, the United States population level estimate of the incidence of T2DM by the Center for Disease Control and Prevention was used (7.8/1000 participant years of follow up).[19] The absolute risk increase associated with T2DM was then calculated using this formula: ARI =
(OR-1)*AI where ARI is the absolute risk increase, OR is the odds ratio and AI is the absolute incidence in events per 1000 participant years.

© 2019 BIRTH-GENE (BIG) Study Working Group. *JAMA Network Open.*

eFigure 1 Schematic representation of a Mendelian randomization approach

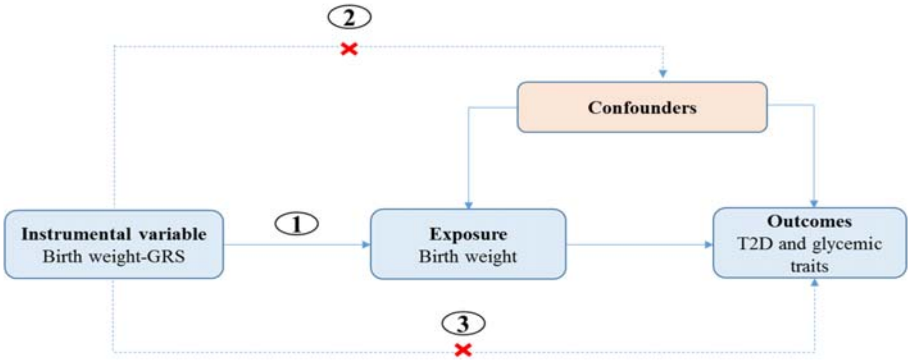

MR can be used to test the hypothesis that exposure (birth weight) causes outcome (T2DM and glycemic traits).
Three assumptions of MR:
1. Genetic variants are associated with birth weight.
2. Genetic variants are not associated with confounders.
3. Genetic variants influence T2D and glycemic traits only through the birth weight, not through other pathways.

eFigure 2 Genetic association with birth weight

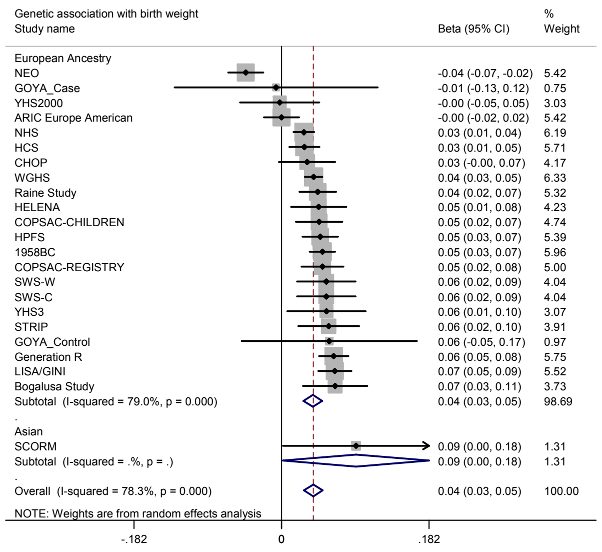

LISA/GINI: LISA/GINIplus.
Results were standardized to a 1-SD decrease in birth weight due to genetic variants.
The genetic risk score for low birth weight was selected as instrumental variable. The lower genetic risk score was associated with higher birth weight. Linear regression models were used to test the association of genetic risk score with birth weight, after adjustment of sex, gestational age and principal components for population stratification region in each study. We pooled β coefficients (1-SD of 543 gram in BW) across 23 studies using random-effect meta-analysis due to the heterogeneity between studies (I^2 =79.2%, P <0.001).

eFigure 3 Genetic association with risk of T2DM

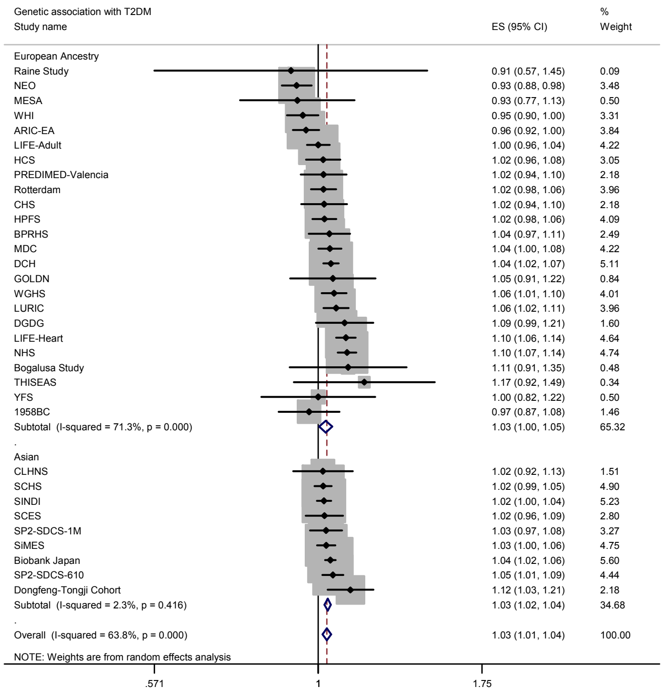

Logistical regression models were used to test the association of genetic risk score with risk of T2DM, after adjustment of gender, gestational age and principal components for population stratification region in each study. We pooled β coefficients across 33 studies using random-effect meta-analysis due to the heterogeneity among studies (I^2 =64.0%, P <0.001).

eFigure 4 Association of birth weight with risk of T2DM

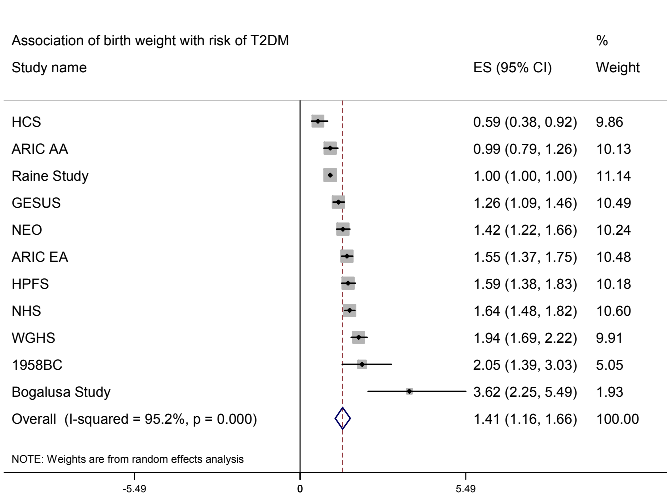

ARIC-AA: ARIC (African Ancestry)
ARIC-EA: ARIC (European Ancestry)
Logistic regression was used to test the association of birth weight with risk of T2DM after adjustment of sex, ethnicity, region, and other baseline covariates if available (age, BMI, smoking status, physical activity, total energy intake, and alcohol intake) in each study.
We pooled OR (1-SD of 543 gram in BW) across 11 studies using random-effect meta-analysis due to the heterogeneity among studies (I^2 = 95.2%, P <0.001).

© 2019 BIRTH-GENE (BIG) Study Working Group. *JAMA Network Open.*

eFigure 5 Causality estimated from individual study.

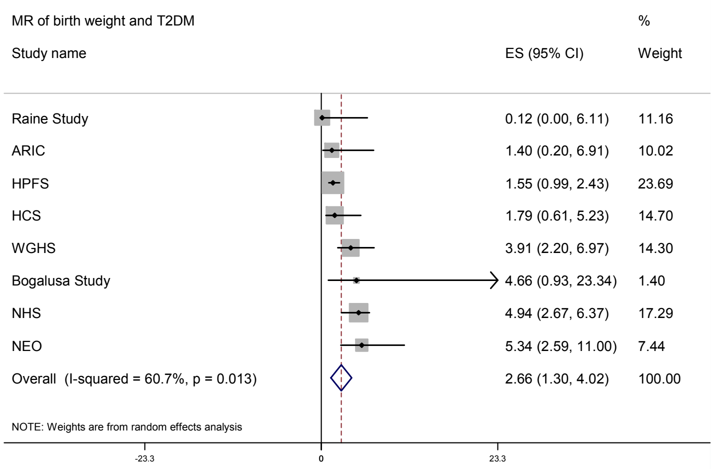

Results were standardized to a 1-SD decrease in birth weight due to genetic risk score. SD is 543 gram from EGG consortium.

We pooled OR across 8 studies that provided both GRS-BW and GRS-T2DM data using random-effect meta-analysis due to the heterogeneity among studies (I^2 =60.7%, P = 0.013).

© 2019 BIRTH-GENE (BIG) Study Working Group. *JAMA Network Open.*

eTable 1. Baseline characteristics of included 49 studies in the CHARGE-BIG study

Full name	Short name	Sample size, n	Study design	Age, years	Baseline year(s)	BMI, kg/m2	Birth weight, kg	Male, n	Male, %	Current Smoker, n	Current Smoker , %	Alcohol intake, g/d	BMI ≥25 kg/m2, n	BMI ≥25 kg/m2, %	diabetes, n	diabetes, %	Ethnicity	Country
Nurses' Health Study	NHS	11194	combined several nested case-control studies	57.9±6.7	1990	26.1±5.1	3.3±0.6	0	0.0	1532	13.7	5.2±9.0	5750	51.4	2177	20.9	European Ancestry	US.
Health Professionals follow-up Study	HPFS	6770	combined several nested case-control studies	55.7±4.8	1994	26.2±3.4	3.3±0.7	6770	100.0	391	5.8	11.6±15.4	4559	67.3	1299	16.9	European Ancestry	US
Diet, Cancer and Health cohort	DCH	3445	Case-cohort (Incident T2D - random cohort)	55.5±4.4	1994-1997	27.3±4.8	NA	1716	49.8	1155	33.6	21.3±23.3	2300	66.8	1,812	52.6	European Ancestry	Denmark
Genetics of Overweight Young Adults-obese case	GOYA-obese	148	Case-cohort (Only the OBESE CASES included in the present analyses)	43.0±6.2	1943-1977	35.6±5.7	3.6±0.7	659	100.0	346	52.5	18.3±29.1	NA	99.2	NA	NA	European Ancestry	Denmark
Genetics of Overweight Young Adults-control	GOYA-control	141	Case-cohort (Only the control included in the present analyses)	43.0±6.2	1943-1977	35.6±5.7	3.6±0.7	659	100.0	346	52.5	18.3±29.1	NA	99.2	NA	NA	European Ancestry	Denmark
Western Australian Pregnancy Cohort (Raine) Study	Raine Study	1688	birth cohort	20.0±0.4	NA	24.6±5.1	3.4±0.7	801	52.0	148	15.6	17.6±19.6	368	34.4	3	0.2	Mixed (Mainly European Ancestry)	Australia
Copenhagen Prospective Studies on Asthma in Childhood Registry	COPSAC-REGISTRY	1191	Asthma exacerbation cases	At birth	1993	NA	3.6±0.5	792	66.4	NA	NA	NA	NA	NA	NA	NA	European Ancestry	Denmark
Copenhagen Prospective Studies on Asthma in Childhood	COPSAC-CHILDREN	932	Birth Cohort	At birth	2007	NA	3.6±0.5	470	50.4	NA	NA	NA	NA	NA	NA	NA	European Ancestry	Denmark
The Danish General Suburban Population Study	GESUS	14169	Cross-sectional	53.7±12.9	2010-2013	26.6±4.6	3.5±0.5	6290	44.4	2468	17.4	12.1±15.1	8352	59.0	794	5.6	European Ancestry	Denmark
Generation R	Generation R	2701	Population-based birth cohort	At birth	0 (birth)	NA	3.6±0.5	1378	51.0	NA	NA	NA	NA	NA	NA	NA	European Ancestry	the Netherlands
Young Finns Study	YFS	1738	longitudinal	37.8±5.0	2007.00	25.8±4.5	NA	774	44.5	328	18.9	8.8±13.6	890	51.2	37.00	2.1	European Ancestry	Finland
Multi-Ethnic Study of Atherosclerosis study	MESA	6361	cohort	62.2±10.2	2000	28.3±5.5	NA	2084	47.3	125	2.0	5.2±13.0	4516	71.0	791	12.5	Mixed (Mainly European Ancestry)	USA
Malmö Diet and Cancer Study	MDC	5040	population based, cohort	57.5±5.9	1992-1996	25.7±4.0	NA	2068	41.0	1379	27.4	10.4±12.6	2615	51.9	985	19.5	European Ancestry	Sweden
Women's Genome Health Study	WGHS	12768	Follow-up	53.4±6.2	1992-1994	25.0±4.3	3.3±0.6	0	0.0	1322	0.1	7.7	5169	0.4	796	0.1	European Ancestry	USA
Hellenic Study of Interactions between SNPs and Eating in Atherosclerosis Susceptibility	THISEAS	1677	CAES-CONTROL	59.1±13.7	NA	28.4±4.7	NA	59	NA	28	NA	8.9±17.5	70	NA	NA	NA	European Ancestry	GREECE
French T2D case-control	DGDG	1279	case-control	56.5±8.7	NA	24.5±2.7	NA	694	50.4	350	25.4	NA	584	42.4	NA	NA	European Ancestry	France
PREvencion con Dieta MEDiterranea-Valencia Study	PREDIMED-Valencia	1023	Prospective cohort (intervention trial)	66.8 ± 6.3	2003-2009	30.6 ± 4.7	NA	371	36.3	129	12.6	5.8±10.4	945	92.4	475	46.4	European Ancestry	Spain
Leipzig Research Centre for Civilization Diseases-Leipzig Heart	LIFE-Heart	5691	patient cohort	62.9±11.3	2006-2014	29.1±4.9	NA	3954	69.5	1492	26.3	NA	4588	80.6	1780	31.3	European Ancestry	Germany
Leipzig Research Centre for Civilization Diseases--Adult	LIFE-Adult	4844	population based, cross-sectional study	62.5±11.1	2012-2014	27.7±4.6	NA	2339	48.3	791	17.4	12.1±17.9	3437	71.0	714	14.8	European Ancestry	Germany
Healthy Lifestyle in Europe by Nutrition in Adolescence	HELENA	802	Cross-sectional	14.7±1.4	NA	21.3±3.8	3.4±0.5	372	46.0	NA	NA	NA	NA	NA	NA	NA	European Ancestry	9 European countries
Genetics of Lipid Lowering Drugs and Diet Network study	GOLDN	819	Prospective cohort	48.8±16.2	NA	28.5±5.5	NA	413	50.4	251	30.7	NA	599	73.1	NA	NA	European Ancestry	USA
Boston Puerto Rican Health Study	BPRHS	1312	Prospective cohort	57.7±7.6	2000	31.9±6.6	NA	378	28.8	321	24.5	1.5±0.6	1131	86.20	NA	NA	Puerto Rican	USA
Southampton Women's Survey (women)	SWS-W	1060	cohort	28.1±3.8	1998-2002	25.4 ±5.0	3.2±0.6	0	0.0	NA	NA	NA	NA	NA	NA	NA	Mixed (Mainly European Ancestry)	UK
Southampton Women's Survey (children)	SWS-C	2005	cohort	At birth	1999-2006	NA	3.5±0.5	1033	52.0	NA	NA	NA	NA	NA	NA	NA	Mixed (Mainly European Ancestry)	UK
German Infant Study on the influence of Nutrition Intervention-LISA/GINI	LISA/GINI	1337	prospective birth cohort studies	At birth	At birth	NA	3.4±0.4	686	51.3	NA	NA	NA	NA	NA	NA	NA	European Ancestry	Germany

© 2019 BIRTH-GENE (BIG) Study Working Group. *JAMA Network Open.*

Singapore Cohort Of the Risk factors for Myopia	SCORM	603	Population-based	NA	NA	NA	NA	NA	NA	NA	NA	NA	NA	NA	NA	NA	Asian	Singapore
Special Turku Coronary Risk Factor Intervention Project	STRIP	674	Prospective randomized intervention study	At birth	NA	NA	3.6±0.4	303	52.7	NA	NA	NA	NA	NA	NA	NA	European Ancestry	Finland
Cebu Longitudinal Health and Nutrition Survey	CLHNS	1798	Population-based	48.5±6.1	2005	24.3±4.4	NA	0	NA	267	14.9	NA	751	42.2	159	8.9	Asian	Philippines
Rotterdam Study	Rotterdam	6291	Prospective cohort	70.6±9.8	1990-1992	29.3±15.1	NA	2509	39.9	1409	22.4	10.4±15.0	3943	62.6	898	24.7	European Ancestry	The Netherlands
Netherlands Epidemiology of Obesity	NEO	5740	Population Based Cohort	56.0±5.9	2008-2012	30.0±4.8	3.3±0.7	2756	48.0	918	16.0	15.5±17.5	5050	88.0	580	10.1	European Ancestry	The Netherlands
European Childhood Obesity Project Study	CHOP	1678	Nutritional Intervention Study (Birth Cohort with 11 years follow up)	At birth	2002-2004	NA	3.2±0.3	850	50.7	NA	NA	NA	NA	NA	NA	NA	Mixed (Mainly European Ancestry)	5 European countries
Women's Health Initiative	WHI	5687	Prospective	68.1±5.9	1993	28.3±5.5	NA	0	0.0	407	15.0	6.0±12.0	3944	69.4	491	8.6	European Ancestry	U.S.
Young Heart Study 2000	YHS2000	306	Cross-sectional	~15	NA	21.4±3.4	3.5±0.5	162	53.0	NA	NA	NA	NA	NA	NA	NA	European Ancestry	Ireland
Young Heart Study III	YHS3	339	Cross-sectional	22.5±1.7	NA	23.8±3.6	3.5±0.5	169	50.0	NA	NA	NA	NA	NA	NA	NA	European Ancestry	Ireland
Dongfeng-Tongji Study	Dongfeng-Tongji Cohort	1452	case-control	63.8±8.1	NA	24.9±3.4	NA	1122	77.3	465	32.0	19.6±1.2	670	46.9	253	17.4	Asian	China
Hertfordshire Cohort Study	HCS	2619	Birth cohort study (born 1931 - 1939)	66.2±2.8	1999-2004	27.4±4.4	3.5±0.5	1467	52.9	343	12.4	10.4±15.8	1927	69.6	389	14.2	European Ancestry	UK
1958 British Birth cohort	1958BC	4040	Cohort study	50.0	1958	27.4±4.9	3.4±0.5	2285	50.3	1047	23.8	66.4±51.1	2972	65.6	151	3.3	European Ancestry	UK
Biobank Japan	Biobank Japan	25332	Case-control	51.8±15.6	NA	22.4±3.7	NA	1723	86.4	NA	NA	NA	NA	NA	1650	NA	Asian	Japan
Ludwigshafen Risk and Cardiovascular Health Study	LURIC	3061	CHD cases and controls	62.7±10.6	1997-2000	27.5±4.0	NA	2144	70.0	709	23.2	16.0±23.9	2196	71.7	1236	40.4	European Ancestry	Germany
Atherosclerosis Risk in Communities Study	ARIC-EA	8415	Perspective Cohort Study	54.3±5.7	1987-1989	27.0±4.8	3.5±0.7	3991	47.4	2085	24.8	10.2±15.8	5285	62.8	2178	25.9	European Ancestry	America
Atherosclerosis Risk in Communities Study	ARIC-AA	2292	Perspective Cohort Study	53.4±5.8	1987-1989	29.9±6.0	3.5±0.8	846	36.9	682	29.8	16.7±26.8	1843	80.4	987	43.1	African American	America
Bogalusa Heart Study	Bogalusa Study	619	cross-sectional studies	41.4±5.6	NA	29.8±7.1	3.4±0.5	292	47.2	190	30.7	NA	457	73.8	41	6.6	European Ancestry	America
Singapore Malay Eye Study	SiMES	2542	Cohort-unrelated	59.1±11.0	NA	26.4±5.1	NA	1258	49.5	529	20.8	NA	1460	57.4	798	31.4	Asian	Singapore
Singapore Indian Eye Study	SINDI	2538	Cohort-unrelated	58.0±10.0	NA	26.2±4.8	NA	1298	51.1	368	14.5	NA	1441	56.8	976	38.5	Asian	Singapore
Diabetic Cohort-Singapore Prospective Study Program	SP2-SDCS-1M	1884	Cohort-unrelated	55.1±13.4	NA	24.1±3.9	NA	1208	64.1	240	12.7	NA	713	37.9	926	49.2	Asian	Singapore
Diabetic Cohort-Singapore Prospective Study Program	SP2-SDCS-610	2232	Cohort-unrelated	56.5±13.3	NA	23.9±4.1	NA	699	30.0	157	7.0	NA	796	35.7	1081	48.4	Asian	Singapore
Singapore Chinese Eye Study	SCES	1889	Population Based Cohort	58.4±9.5	NA	23.7±3.5	NA	963	51	246	13	NA	600	1289	287	15	Asian	Singapore
Cardiovascular Health Study	CHS	2820	prospective	72.3±5.4	1989-1990	26.0±4.3	NA	1066	37.8	323	11.5	5.7±13.1	1585	56.2	258	NA	European Ancestry	USA
Singapore Chinese Health Study	SCHS	4570	Case-control (T2D case-control)	55.7±7.4	NA	23.7±3.4	NA	2423	53.0	1233	27.0	1.8±7.7	1271	27.8	2281	NA	Asian	Singapore

Data for age, BMI, and birth weight are expressed as mean +/- SD.

© 2019 BIRTH-GENE (BIG) Study Working Group. *JAMA Network Open*.

eTable 2. Assessment of birth weight and covariates in the CHARGE-BIG study

	Dietary assessment		Birth weight, kg			BMI, kg/m²			Exclulsion criteria	Software for analysis	All participants provided written, informed consent, and ethical approval was granted by local ethics committees for participating studies.
	Measurement (FFQ/diet record/ diet recall/etc)	Time of assessment	Mean ±SD	Clinical measurement/ self-reported	Time of measurement	Mean ±SD	Clinical measurement/ self-reported	Time of measurement			
NHS	FFQ	1990	3.3 ± 0.6	Self-reported	1992	26.1 ± 5.1	Self-reported	1990	Exclude cancer cases	SAS	Yes
HPFS	FFQ	1994	3.3 ± 0.7	Self-reported	1994	26.2 ± 3.4	Self-reported	1994	Exclude cancer cases	SAS	Yes
DCH	FFQ	1994-1997	NA	NA	NA	27.3 ± 4.8	Measured	1994-1997	NA	SAS	Yes
GOYA obese cases	NA	NA	NA	NA	NA	35.6 ± 5.7	Measured	1992-1994	NA	SAS	Yes
Raine	FFQ	2010-2012	3.4 ± 0.5	Clinical measurement	1989-1991	24.5 ± 5.2	Clinical measurement	2010-2012	NA	R	Yes
COPSAC REGISTRY	NA	NA	3.5 ± 0.5	Self-reported	1993	NA	NA	NA	Excluded based on incomplete genetic or phenotypic data or non notmal gestational age	R	Yes
COPSAC	NA	NA	3.5 ± 0.5	Self-reported	2007	NA	NA	NA	Excluded based on incomplete genetic or phenotypic data or non notmal gestational age	R	Yes
WGHS	FFQ	1992-1995	3.3 ± 0.6	Self-reported	1992-1995 (baseline)	25.0 ± 4.3	Clinical measurement	2010-2013	NA	Stata	Yes
GESUS	NA	NA	NA	NA	NA	NA	NA	NA	NA	NA	NA
Generation R	NA	NA	3.6±0.5	Clinical measurement	2002-2006	NA	NA	NA	NA	SPSS	Yes
YFS	FFQ	2007	-	NA	NA	25.8 ± 4.5	Clinical measurement	2007	-	R	Yes
MESA	NA	NA	NA	NA	NA	NA	NA	NA	NA	NA	NA
MDC	FFQ	1991-1994	NA	NA	NA	26.1 ± 5.1	Clinical measuremen	1992-1994	NA	SPSS	Yes
THISEAS	FFQ	2010	NA	NA	NA	29.2 ± 4.5	Clinical measuremen	2010	Ethnic outliers,implausible energy intake	R	Yes
DGDG	FFQ	1994-2004	NA	NA	NA	24.5 ± 2.7	Clinical measuremen	NA	NA	R	Yes
PREDIMED-Valencia	FFQ	2003-2009	N/A	N/A	NA	30.6 ± 4.7	Clinical Mesured	2003-2009	Exclude baseline CVD cases and cancer cases	SPSS	Yes
LIFE-Heart	NA	NA	NA	NA	NA	29.1 ± 4.9	Measurement	2006-2014	Age<18y	R	Yes
LIFE-Adult	FFQ	2012-2014	NA	NA	NA	27.7 ± 4.6	Measurement	2012-2014	Age<18y	R	Yes
HELENA	NA	NA	3.4 ± 0.5	Self-reported	2006-2007	21.3 ± 3.8	Clinical measurement	2006-2007	Exclude preterm births (gestational age<37 weeks)	SAS	Yes

© 2019 BIRTH-GENE (BIG) Study Working Group. *JAMA Network Open.*

GOLDN	NA	NA	NA	NA	NA	NA	NA	NA	NA	NA	NA
BPRHS	NA	NA	NA	NA	NA	NA	NA	NA	NA	NA	NA
SWS-W	NA	NA	3.2 ± 0.6	Self-reported	1998-2002	25.4 ± 5.0	Clinical measurement	1998-2002	Multiple births	Stata	Yes
SWS-C	NA	NA	3.5 ± 0.5	Clinical measurement	1999-2006	NA	-	-	Pre-term births (multiple births excluded by design)	Stata	Yes
LISA/GINI	NA	NA	NA	NA	NA	NA	NA	NA	NA	NA	NA
SCORM	NA	NA	NA	NA	NA	NA	NA	NA	NA	NA	NA
STRIP	NA	NA	3.6 ± 0.5	Clinical records of the well-baby clinics	1989-1991	NA	NA	NA	Gestational age less than 37 weeks	R	Yes
CLHNS	NA	NA	NA	NA	NA	24.3 ± 4.4	Clinical measurement	2005	NA	R	Yes
Rotterdam	FFQ	1989-1993	NA	NA	NA	29.3 ± 15.1	Clinical measurement	1989-1993	NA	SPSS	Yes
NEO	FFQ	2008-2012	4.3 ± 0.7	Self-reported	2008-2012	30.0 ± 4.8	Clinical measurement	2008-2012	None	SPSS	Yes
CHOP	NA	NA	3.3 ± 0.3	Clinical measurement	2002-2004	NA	NA	NA	born <37th gestational week	SAS / R	Yes
WHI	NA	NA	NA	NA	NA	NA	NA	NA	NA	NA	NA
YH2000	NA	NA	3.5 ± 0.5	Department of Health and Social Services	2000-2001	21.4 ± 3.4	Clinical measurement	2000-2001	Exclude multiple births and preterm births (gestational age<37 weeks)	SAS	Yes
YH3	NA	NA	3.5 ± 0.5	Department of Health and Social Services	1997-1999	23.8 ± 3.6	Clinical measurement	1997-1999	Exclude multiple births and preterm births (gestational age<37 weeks)	SAS	Yes
DF-TJ	NA	NA	NA	NA	NA	NA	NA	NA	Exclusion criteria included chemotherapy or radiotherapy for cancer and other severe diseases	SAS	Yes
HCS	FFQ	1999 - 2004	3.45 ± 0.51	Clinical measuremen	1931 - 1939	27.4 ± 4.3	Clinical measurement	1999 - 2004	Exclude pre-term births (gestational age <37 weeks)	Stata	Yes
1958BC	FFQ	2000	3.4 ± 0.5	Clinical measurement	1958	27.4 ± 4.9	Clinical measurement	2002/3 (45 years biomedical survey)	None	Stata	Yes
Biobank Japan	-	-	-	-	-	22.8 ± 3.6	Medical records	NA	The exclusion criteria for cases were individuals positive for antibody to glutamic acid decarboxylase (GAD) or those with diabetes due to liver dysfunction, steroids and other drugs that might raise glucose levels, malignancy or monogenic disorder known to cause diabetes.	R (v3.1.3)	Yes
LURIC	NA	NA	NA	NA	NA	27.5 ± 4.03	Measurement	1997-2000	Missing genotype data	SPSS	Yes
ARIC Europe American	FFQ	NA	3.5 ± 0.7	Self-reported	visit 4(1996-1998)	27.0 ± 4.9	Clinical measurement	End of follow-up	NA	Stata	Yes
ARIC African American	FFQ	NA	3.5 ± 0.8	Self-reported	visit 4(1996-1998)	30.0 ± 6.2	Clinical measurement	End of follow-up	NA	Stata	Yes
Bogalusa Study	NA	NA	3.4 ± 0.5	Self-reported	1973-1982	29.8 ± 7.1	Clinical measurement	2008	Excluding multiple births and, where information was available, preterm births (gestational age<37 weeks) from all analyses	R	Yes
SiMES	NA	NA	NA	NA	NA	0.56± 0.50	Clinical measurement	2004	NA	R	Yes
SINDI	NA	NA	NA	NA	NA	26.1± 4.8	Clinical measurement	2007	NA	R	Yes

© 2019 BIRTH-GENE (BIG) Study Working Group. *JAMA Network Open.*

DC-SP2-1M	NA	NA	NA	NA	NA	24.1± 3.8	Clinical measureme nt	2003-2007	NA	R	Yes
DC-SP2-610	NA	NA	NA	NA	NA	23.9± 4.1	Clinical measureme nt	2003-2007	NA	R	Yes
SCES	NA	NA	NA	NA	NA	23.7± 3.5	Clinical measureme nt	2007	NA	R	Yes
CHS	FFQ	1989-1990	NA	NA	NA	26.0 ± 4.3	Clinical measureme nt	1989-1990	Exclude baseline diabetes	Stata	Yes
SCHS	FFQ	?	NA	NA	NA	22.9 ± 3.4	Self-reported	NA	NA	Stata	Yes

eTable 3. Assessment of type 2 diabetes in the CHARGE-BIG study

	Outcome	Mesurement time point of disease			
		Incident/prevalent/incident+prevale nt	Validation	Self-reported/medical records/defined by researcher	The way how it was defined by researcher
NHS	Diabetes	Incident+prevalent disease	Validated	Self-reported and validated	ICD-8 was used in validation study
HPFS	Diabetes	Incident+prevalent disease	Validated	Self-reported and validated	ICD-8 was used in validation study
DCH	Diabetes	Incident disease	Validated	Medical records	NA
GOYA	Birth weight	Birth weight	NA	Medical records	NA
Raine	Diabetes	Incident +prevalent disease	Validated	Self-reported and validated	Usage of medication, diagnosed with diabetes by a doctor, fasting glucose > 7mmol/L after excluding ICD-9 T1D
COPSAC REGISTRY	Birth weight	NA	NA	Medical records	NA
COPSAC	Birth weight	NA	NA	Medical records	NA
GESUS	Diabetes	NA	Self-reported	Self-reported	NA
Generation R	Birth weight	Birth weight	NA	Medical records	NA
YFS	Diabetes	Incident+prevalent disease	Validated	Medical records	NA
MESA	NA	NA	NA	NA	NA
MDC	Diabetes	Incident+prevalent disease	Validated	National and regional registers and cohort-measurements	Diagnosis by physician (if-glucose >=7.0 mmol/L measured twice, or at least two HbA1c >6.9%, or ICD-10 codes E10-E14 and O244-0249, or ATC code A10 medication
WGHS	Diabetes	Prevalent disease	Self-reported	Self-reported	NA
THISEAS	Diabetes	Prevalent disease	medical records	NA.	NA
DGDG	Diabetes	Prevalent disease	NA	NA	NA
PREDIMED-Valencia	Diabetes	Prevalent disease	Validated	Medical records	Validated
LIFE-Heart	Diabetes	Prevalent disease	Validated	Self-reported and defined by researcher	anamnestic diabetes OR diabetes medication OR HbA1c>=6.5%
LIFE-Adult	Diabetes	Prevalent disease	Validated	Self-reported and defined by researcher	anamnestic diabetes OR diabetes medication OR HbA1c>=6.5%
HELENA	Birth weight	NA	NA	Self-reported	NA

© 2019 BIRTH-GENE (BIG) Study Working Group. *JAMA Network Open.*

GOLDN	Diabetes	Incident+prevalent disease	Self-reported	Self-reported	NA
BPRHS	Diabetes	Incident+prevalent disease	Self-reported	Self-reported	NA
SWS-C	Birth weight	-	NA	N/A	NA
LISA/GINI	Birth weight	NA	NA	NA	NA
SCORM	Birth weight	NA	Validated	Medical records	NA
CLHNS	Diabetes	Prevalent disease	Validated	Medical record + defined by researcher	NA
RS	Diabetes	Incident+prevalent disease	Validated	Medical records and clinical measurements	Fasting blood glucose ≥7.0 mmol/L, a non-fasting blood glucose ≥11.1 mmol/L (when fasting samples were absent), or the use of blood glucose lowering medication
NEO	Diabetes	Prevalent disease	Validated	Self-reported and validated	Self-reported, use of glucose-lowering medication or fasting glucose ≥7.0 mmol/L
CHOP	Birth weight	NA	NA	Medcial records	NA
WHI	Diabetes	Incident+prevalent disease	NA	Medical records	NA
YH2000	Birth weight	NA	NA	Department of Health and Social Services	NA
YH3	Birth weight	NA	NA	Department of Health and Social Services	NA
DF-TJ	Diabetes	Prevalent disease	Validated	Self-reported and validated	ICD-9 was used in validation study
HCS	Diabetes	Prevalent disease	Validated	Self-reported & OGTT	NA
1958BC	Diabetes	Prevalent disease	Self-reported	Self-reported	NA
BBJ	Diabetes	Prevalent disease	Validated	Defined by researcher	Diagnosed according to the WHO criteria.
LURIC	Diabetes	Prevalent disease	Validated	Self-reported, 2H-oGTT, HbA1c measurement	NA
ARIC	Diabetes	Incident+prevalent disease	Validated	Self-reported and validated by blood work and medication status	fasting glucose levels, taking medications for diabetes
Bogalusa Study	Diabetes	Incident disease	Validated	Self-reported/medical records	NA.
SiMES	Diabetes	Prevalent disease	Validated	Defined by researcher	A person is classified as diabetes if his/her hba1c>=6.5 or on anti-diabetic drug or with age of diagnosis
SINDI	Diabetes	Prevalent disease	Validated	Defined by researcher	A person is classified as diabetic if his/her hba1c>=6.5 or on anti-diabetic drug or with age of diagnosis
DC-SP2	Diabetes	Prevalent disease	Validated	Defined by researcher	A person is classified as diabetic if his/her is on anti-diabetic medication or with fasting glucose >7
SCES	Diabetes	Prevalent disease	Validated	Defined by researcher	A person is classified as diabetes if his/her hba1c>=6.5 or on anti-diabetic drug
CHS	Diabetes	Incident disease	Validated	Based on inventory of medications and fasting glucose	NA
SCHS	Diabetes	Incident disease	NA	Medical records	NA

© 2019 BIRTH-GENE (BIG) Study Working Group. *JAMA Network Open*.

eTable 4. Genotyping information in the CHARGE-BIG study

Short name	Disease outcome	Genetic risk score1 (GRS1)			Genotyping method		CCNL1 (rs900400)	ADCY5 (rs9883204)	HMGA2 (rs1042725)	CDKAL1 (rs6931514)	5q11.2 (rs4432842)	LCORL (rs724577)	ADRB1 (rs1801253)	All participants provided written, informed consent, and ethical approval was granted by local ethics committees for participating studies.
		Number of SNP included in analysis	Median (IQR)	Range (min, max)	Genotyping method	Sample call rate	EAF/HWE-Pvalue	EAF/ HWE-Pvalue	EAF/ HWE-Pvalue	EAF/ HWE-Pvalue	EAF/ HWE-Pvalue	EAF/ HWE-Pvalue	EAF/ HWE-Pvalue	
NHS	T2D	7	6.6 (2.5)	(0.7, 13.2)	TaqMan	97%	0.42/0.003	0.76 /0.85	0.49/0.38	0.26/ 0.07	0.29/0.27	0.73/0.57	0.29/0.71	Yes
HPFS	T2D	7	6.7 (2.5)	(0.8, 12.3)	TaqMan	97%	0.43/0.82	0.76/0.33	0.51/0.85	0.26/0.77	0.27/0.09	0.75/0.45	0.31/0.90	Yes
DCH	T2D	7	6.4 (2.5)	(0.9, 11.7)	Illumina HumanCoreExome BeadChip	>95%	0.41/0.75	0.73/0.25	0.47/0.92	0.28/0.51	0.20/0.51	0.73 /0.25	0.26/0.11	Yes
GOYA	Birth weight	7	6.4 (2.4)	(1.8, 11.5)	Illumina 610 k quad chip	>95%	0.42/0.81	0.74/0.10	0.48/0.69	0.30/0.33	0.21/0.44	0.74/0.60	0.26/0.97	Yes
Raine	T2D	7	6.4 (2.3)	(1.7, 12.0)	Illumina	97%	0.39/0.39	0.76/0.83	0.46/0.64	0.27/0.9	0.21/ 0.04	0.74/0.84	0.26/ 0.59	Yes
COPSAC REGISTRY	Birth weight	7	6.4 (2.5)	(0,85 12.4)	Illumina SNP array	95%	0.41/0.43	0.71 /0.79	0.47/0.22	0.28/0.035	0.31/0.87	0.73/0.55	0.25/0.20	Yes
COPSAC CHILDREN	Birth weight	7	6.4 (2.4)	(1.5, 12.3)	Illumina SNP array	95%	0.40/0.04	0.73 /0.12	0.47/0.93	0.31/0.21	0.30/0.26	0.71/0.46	0.25/0.40	Yes
GESUS	T2D	7	NA	NA	NA	NA	NA	NA	NA	NA	NA	NA	NA	Yes
Generation R	Birth weight	7	6.4 (2.4)	(0.8,12.9)	GWAS, Illumina 610 Quad and 660 platforms	98%	0.41/>1e-6	0.71/>1e-6	0.49/>1e-6	0.27/>1e-6	0.30/>1e-6	0.72/>1e-6	0.24/>1e-6	Yes
YFS	T2D	7	4.3 (2.4)	(0.9, 10.8)	custom Illumina BeadChip Human670K	95%	0.39/0.78	0.22/0.73	0.61/0.43	0.44/0.82	0.41/0.71	0.40/0.50	0.32/0.99	Yes
MESA	T2D	7	NA	NA	NA	NA	NA	NA	NA	NA	NA	NA	NA	Yes
MDC	T2D	7	6.5 (2.4)	(0.7, 12.6)	Illumina HumanOmniExpress BeadChip	100%	0.42/0.67	0.74 /0.10	0.47/0.60	0.27/0.04	0.31/0.20	0.71/0.43	0.27/0.85	Yes
WGHS	T2D	7	6.6(5.4-7.8)	(0.02,13.2)	Illumina HumanHap300 Duo "+"	>90%	0.41/0.40	0.75/0.99	0.49/0.98	0.26/0.96	0.29/0.47	0.73/0.99	0.28/0.90	Yes
THISEAS	T2D	7	NA	NA	NA	NA	NA	NA	NA	NA	NA	NA	NA	Yes
DGDG	T2D	7	6.8 (2.5)	(1.2, 12.5)	Illumina HAP300 or imputation (IMPUTE2 certainty >0.98, calling if probability>0.8)	97%	0.42/0.06	0.76/0.07	0.52/1.00	0.26/0.14	0.27/0.45	0.73/0.29	0.26/0.32	Yes
PREDIMED-Valencia	T2D	7	5.82	(1.46, 12.09)	Illumina Omni Express array	97%	0.41/0.529	0.82/0.784	0.57/0.554	0.14/0.791	0.17/0.175	0.60/0.053	0.17/0.173	Yes
LIFE-Heart	T2D	7	6.8 (5.5-8.0)	(0.9,13.0)	Affymetrix Axiom-CADLIFE array + Imputation	97%	0.44/0.96	0.77/0.23	0.48/0.89	0.28/0.22	0.29/0.61	0.74/0.63	0.27/0.86	Yes
LIFE-Adult	T2D	7	6.8 (5.5-8.0)	(0.9,13.0)	Affymetrix Axiom-CEU SNP array + Imputation	97%	0.44/0.64	0.77/0.71	0.48/0.71	0.29/0.70	0.29/0.21	0.74/0.077	0.27/0.97	Yes
HELENA	Birth weight	6	6.3 (2.2)	(1.7, 10.6)	KASPAR	100%	0.44/0.26	0.80/0.98	0.55/0.0007	0.26/0.06	0.28/0.33	0.75/0.60	NA	Yes
GOLDN	T2D	7	NA	NA	NA	NA	NA	NA	NA	NA	NA	NA	NA	Yes
BPRHS	T2D	7	NA	NA	NA	NA	NA	NA	NA	NA	NA	NA	NA	Yes
SWS-W	Birth weight	6	6.0 (2.2)	(0.8, 12.0)	KASP PCR-based (LGC)	≥97%	0.41/0.76	0.75/0.82	0.51/0.52	0.25/0.92	0.31/0.34	0.74/0.55	Failed to genotype whole sample	Yes

© 2019 BIRTH-GENE (BIG) Study Working Group. *JAMA Network Open.*

SWS-C	Birth weight	6	5.9 (2.3)	(1.5, 11.3)	KASP PCR-based (LGC)	≥97%	0.39/0.10	0.74/0.41	0.51/0.13	0.26/0.61	0.31/0.35	0.75/0.03	Failed to genotype whole sample	Yes
LISA/GINI	Birth weight	7	6.7 (2.6)	(0.9, 12.1)	Affy5/Affy6 (those with 100% genotyped; <100% imputed)	⩾98%	0.42/0.09	0.78/0.76	0.5/1	0.27/0.90	0.29/0.76	0.74/0.95	0.27/0.27	Yes
SCORM	Birth weight		NA	NA	NA	NA	NA	NA	NA	NA	NA	NA	NA	Yes
STRIP	Birth weight	7	6.41 (2.31)	(2.15, 11.77)	Metabochip	90%	0.337/0.93	0.823/0.582	0.466/0.235	0.317/0.450	0.325/0.877	0.647/0.223	0.243/0.665	Yes
CLHNS	T2D	7	8.3 (1.0)	(3.2, 13.3)	Affy 5.0	97%	0.484/0.57	0.993/0.0018	0.822/0.60	0.408/0.61	0.377/0.21	0.680/0.89	0.255/0.98	Yes
Rotterdam	T2D	7	7.1 (2.5)	(0.9, 13.2)	Illumina Infinium Human Hap 550K	>95%	0.411/0.947	0.720/0.825	0.482/0.994	0.264/0.842	0.300/0.967	0.727/0.933	0.261/0.842	Yes
NEO	T2D	7	6.37 (2.41)	12.16 (0.85, 13.01)	Illumina HumanCoreExome chip	100%	0.41/0.40	0.72/0.44	0.46/0.27	0.27/0.30	0.29/0.84	0.71/0.20	0.26/0.95	Yes
CHOP	BW	7	7.8 (2.2)	(1.9, 12.3)	Human OmniExpress-24 v1.0	97%	0.44/0.535	0.81/ 0.328	0.57/0.038	0.72/0.898	0.26/0.894	0.73/ 0.092	0.29/ 0.902	Yes
WHI	T2D	7	NA	NA	NA	NA	NA	NA	NA	NA	NA	NA	NA	Yes
YH2000	Birth weight	6	5.7 (2.2)	(2, 9.7)	KASPAR	100%	0.34/0.019	0.71/0.29	0.47/0.27	0.26/0.69	0.33/0.45	0.75/0.41	NA	Yes
YH3	Birth weight	6	5.8 (2.3)	(1.2, 9.7)	KASPAR	100%	0.36/0.78	0.76/0.14	0.44/0.018	0.25/0.36	0.34/0.20	0.74/0.13	NA	Yes
DF-TJ	T2D	7	8.4 (2.3)	(3.1, 13.2)	Affymetrix 6.0	99.4%	0.52/0.60	1.00/0.93	0.80/0.03	0.50/0.10	0.42/0.91	0.51/0.57	0.24/0.45	Yes
HCS	T2D	6	6.0 (2.3)	(0.7, 11.2)	KASP Allele specific PCR	100%	0.40/0.97	0.73/0.81	0.51/0.94	0.27/0.94	0.29/0.97	0.74/0.90	NA	Yes
1958BC	T2D	7	6.5 (2.5)	(0.9, 12.2)	Affymetrix 6.0 or Illumina Infinium 550K.	≥ 95%	0.39/0.78	0.73/0.20	0.50/0.55	0.27/0.08	0.31/0.71	0.74 /0.75	0.24/0.09	Yes
Biobank Japan	T2D	7	7.88 (2.33)	(2.40, 13.34)	Imputation (Cases and controls were genotyped by Illumina Human610-Quad BeadChip)	100% (imputation)	0.496/-	1.000/-	0.726/-	0.467/-	0.365/-	0.505/-	0.193/-	Yes
LURIC	T2D	7	6.8 (2.4)	(1.7, 12.3)	Microarray	100%	0.42/0.45	0.77/0.68	0.48/0.88	0.26/0.56	0.27/1.00	0.76/1.00	0.28/0.72	Yes
ARIC-EA	T2D	7	7.5 (2.4)	(1.5, 13.3)	Imputed	NA	0.41/0.9313	0.75/0.9494	0.49/0.9996	0.27/0.9926	0.29/0.9997	0.74/0.9985	0.27/0.9743	Yes
ARIC-AA	T2D	7	7.5 (2.3)	(1.3, 12.5)	Imputed	NA	0.25/0.9805	0.66/0.8796	0.38/0.9992	0.24/0.9924	0.75/1.000	0.71/0.9850	0.42/0.8552	Yes
Bogalusa Study	T2D	7	6.1 (2.3)	(0.7, 12.0)	Illumina Human610 BeadChip	NA	0.41/0.079	0.78/0.238	0.50/0.080	0.28/0.835	0.22/0.726	0.76/0.507	0.27/0.923	Yes
SiMES	T2D	5	6.1(3.0)	(0,13.004)	Illumina 610Quad	95%	0.410264/0.4	NA	NA	0.43922/0.243	0.319954/0.4747	0.712729/0.3485	0.275803/0.5488	Yes
SINDI	T2D	4	5.3(3.5)	(0,12.6)	Illumina 610Quad	95%	0.247119/0.7342	NA	NA	0.246799/0.9458	NA	0.867798/0.1217	0.25128/0.1784	Yes
DC-SP2-1M	T2D	6	7.3(2.7)	(1.9,13.1)	Illumina 1M duov3	95%	0.534447/0.3633	NA	0.822547/0.9118	0.455637/0.948	0.425887/0.6441	0.577244/0.0009091	0.253653/0.8644	Yes
DC-SP2-610	T2D	5	6.3(3.1)	(0,13.4)	Illumina 610Quad	95%	0.538662/0.5534	NA	NA	0.459166/0.1728	0.383579/0.8517	0.573414/1	0.254996/0.7563	Yes
SCES	T2D	5	6.531(3.193)	(0,13.004)	Illumina 610Quad	0.95	0.540262/0.002532	NA	NA	0.475655/0.3399	0.396692/0.9169	0.567104/0.6111	0.252497/1	Yes
CHS	T2D	7	6.8 (2.5)	0.9, 12.6	Illumina HumanCNV370-Duo BeadChip and imputation to HapMap		0.42	0.75	0.52	0.27	0.3	0.74	0.29	Yes
SCHS	T2D	6	6.7(3.1)	(0,12.4)	Illumina 610Quad	95%	NA	NA	NA	NA	NA	NA	NA	Yes

© 2019 BIRTH-GENE (BIG) Study Working Group. *JAMA Network Open.*

eTable 5. Distribution of genotypes of included 7 SNPs in the CHARGE-BIG study

	rs900400			rs9883204			rs1042725			rs6931514			rs4432842			rs724577			rs1801253		
**Short name**	**CCNL1_0, %**	**CCNL1_1, %**	**CCNL1_2, %**	**ADCY5_0, %**	**ADCY5_1, %**	**ADCY5_2, %**	**HMGA2_0, %**	**HMGA2_1, %**	**HMGA2_2, %**	**CDKAL1_0, %**	**CDKAL1_1, %**	**CDKAL1_2, %**	**5q11.2_0, %**	**5q11.2_1, %**	**5q11.2_2, %**	**LCORL_0, %**	**LCORL_1, %**	**LCORL_2, %**	**ADRB1_0, %**	**ADRB1_1, %**	**ADRB1_2, %**
NHS	34.9	47.5	17.6	6.0	37.0	57.0	25.6	49.8	24.7	53.1	39.2	7.7	50.0	41.1	8.9	7.1	38.2	54.8	52.3	40.2	7.5
HPFS	33.6	48.7	17.7	6.2	36.1	57.7	23.4	49.1	27.5	52.8	39.9	7.3	53.6	38.1	8.3	6.3	37.9	55.8	51.2	39.9	8.9
DCH	35.7	48.4	16.0	7.4	37.6	55.0	27.6	49.8	22.6	50.1	41.9	8.0	63.3	32.4	4.3	6.7	40.2	53.2	54.1	39.5	6.4
GOYA	37.3	45.7	17.0	7.9	35.8	56.3	29.3	44.8	26.0	50.8	42.2	7.0	65.1	30.4	4.6	6.4	41.3	52.4	53.9	40.2	5.9
Raine Study	35.8	48.8	15.4	6.0	37.3	56.7	27.7	49.0	23.3	54.7	38.0	6.6	64.4	30.5	5.2	6.5	38.0	55.5	55.3	37.7	7.0
COPSAC-REGISTRY	35.4	47.3	17.3	8.4	40.6	51.0	27.5	51.6	21.0	50.6	42.8	6.6	47.3	42.8	9.9	7.2	40.6	52.2	56.6	36.9	6.5
COPSAC-CHILDREN	34.0	51.4	14.6	6.4	41.6	51.9	27.6	50.0	22.4	46.9	44.4	8.7	48.2	43.6	8.3	8.7	39.9	51.4	55.9	38.4	5.7
GESUS	NA	NA	NA	NA	NA	NA	NA	NA	NA	NA	NA	NA	NA	NA	NA	NA	NA	NA	NA	NA	NA
Generation R	35.8	46.6	17.6	8.7	39.5	51.8	26.2	49.4	24.4	53.5	39.1	7.4	48.9	41.3	9.8	7.9	40.2	51.9	56.9	36.8	6.3
YFS	48.2	42.4	9.4	65.5	30.7	3.8	28.8	49.0	22.2	44.7	44.7	11.7	46.7	43.2	10.1	46.4	42.6	11.1	54.7	38.8	6.6
MESA	37.7	46.1	16.2	7.8	34.7	57.5	23.1	46.9	30.1	51.6	39.0	9.4	31.8	41.8	26.4	10.1	42.2	47.7	9.6	41.2	49.2
MDC	36.1	47.7	16.2	6.6	38.1	55.3	28.7	49.9	21.4	52.4	39.8	7.8	47.8	42.8	9.3	7.7	40.0	52.2	53.6	38.8	7.6
WGHS	34.1	48.7	17.2	6.1	37.7	56.1	25.9	49.5	24.6	54.4	38.6	7.0	49.5	41.9	8.7	7.2	38.6	54.3	52.4	39.9	7.6
THISEAS	30.8	48.3	20.9	3.3	32.7	63.9	14.8	47.4	37.8	53.1	39.2	7.7	53.7	38.6	7.7	4.4	32.8	62.8	49.1	41.3	9.6
DGDG	34.4	48.3	17.3	6.6	32.7	60.7	20.9	49.7	29.4	52.1	37.3	10.5	52.9	39.5	7.6	5.5	39.9	54.6	50.2	39.8	7.0
PREDIMED	34.7	49.2	16.1	3.5	29.8	66.6	18.4	50.0	31.5	73.2	24.8	2.0	68.5	29.2	2.2	14.6	50.9	34.5	68.5	29.3	2.3
LIFE-Heart	30.9	49.4	19.7	5.6	34.9	59.5	27.0	50.0	29.9	51.6	40.0	8.4	49.9	41.3	8.8	6.7	37.9	55.4	54.1	38.9	7.1
LIFE-Adult	31.4	48.9	19.6	5.2	34.9	59.8	26.7	50.3	23.0	50.2	41.5	8.3	49.7	42.1	8.2	7.2	37.2	55.6	52.8	39.7	7.5
HELENA	33.0	48.0	19.0	4.0	32.0	64.0	23.0	44.0	33.0	54.0	40.0	6.0	51.0	42.0	7.0	6.0	37.0	57.0	NA	NA	NA
GOLDN	37.5	46.6	15.9	60.1	34.1	5.9	26.9	47.0	26.1	8.1	43.1	48.8	51.2	40.3	8.5	55.6	37.4	7.1	6.0	42.7	51.3

© 2019 BIRTH-GENE (BIG) Study Working Group. *JAMA Network Open.*

BPRHS	17.1	43.9	39.1	53.1	39.3	7.6	19.1	48.6	32.2	57.5	35.0	7.5	28.6	44.2	27.2	9.7	41.7	48.6	48.6	41.5	9.9
SWS-W	35.0	48.0	17.0	6.0	38.0	56.0	23.0	51.0	26.0	56.0	38.0	6.0	49.0	42.0	10.0	6.0	38.0	56.0	57.0	38.0	4.0
SWS-C	38.0	46.0	16.0	6.0	39.0	54.0	23.0	52.0	26.0	54.0	39.0	7.0	48.0	42.0	10.0	5.0	40.0	55.0	57.0	36.0	7.0
LISA/GINI	35.0	46.3	18.7	4.6	34.5	60.9	25.8	49.7	24.5	53.4	39.0	7.6	49.8	41.6	8.6	7.3	38.3	55.0	52.1	40.6	7.3
SCORM	NA	NA	NA	NA	NA	NA	NA	NA	NA	NA	NA	NA	NA	NA	NA	NA	NA	NA	NA	NA	NA
STRIP	43.8	43.8	11.3	2.8	29.7	67.5	29.7	47.3	23.0	45.9	44.7	9.4	45.4	44.2	10.4	11.3	48.0	40.7	57.6	36.2	6.3
CLHNS	27.0	49.3	23.7	0.1	1.3	98.7	3.3	28.9	67.8	34.8	47.9	17.3	38.0	48.4	13.6	10.1	43.6	46.3	55.5	38.0	6.5
Rotterdam	16.3	49.7	34.0	7.7	40.4	51.9	26.8	50.0	23.2	7.0	39.0	54.0	8.9	42.1	49.0	7.2	40.2	52.6	6.9	38.5	54.7
NEO	34.7	49.1	16.2	7.5	41.0	51.5	28.2	50.8	21.0	53.8	38.5	7.6	49.7	41.8	8.5	8.8	40.1	51.0	55.5	37.9	6.6
CHOP	30.4	51.2	18.4	2.9	33.4	63.7	16.1	54.7	29.3	7.3	40.7	52.1	55.2	37.8	7.0	8.8	36.0	55.2	50.7	41.3	8.1
WHI	NA	NA	NA	NA	NA	NA	NA	NA	NA	NA	NA	NA	NA	NA	NA	NA	NA	NA	NA	NA	NA
YHS2000	39.0	53.0	8.0	8.0	41.0	51.0	26.0	54.0	20.0	55.0	40.0	5.0	44.0	44.0	12.0	6.0	39.0	55.0	NA	NA	NA
YHS3	41.0	47.0	13.0	5.0	38.0	57.0	28.0	54.0	18.0	56.0	39.0	5.0	41.0	47.0	12.0	9.0	33.0	58.0	NA	NA	NA
Dongfeng-Tongji Cohort	22.5	49.1	27.4	0.0	0.8	99.2	3.2	31.8	65.1	22.0	52.3	23.0	32.3	48.9	18.7	23.9	50.1	25.5	53.2	40.0	6.8
HCS	34.8	49.2	16.0	7.7	37.7	54.6	24.2	49.8	26.0	52.7	40.2	7.1	51.0	40.6	8.4	5.8	40.1	54.1	NA	NA	NA
1958BC	37.3	47.4	15.3	7.1	40.6	52.2	25.2	50.4	24.3	53.6	38.5	7.9	47.6	42.9	9.4	6.6	37.8	55.7	57.3	37.4	5.3
Biobank Japan	NA	NA	NA	NA	NA	NA	NA	NA	NA	NA	NA	NA	NA	NA	NA	NA	NA	NA	NA	NA	NA
LURIC	33.0	48.1	19.0	5.3	36.1	58.5	24.1	51.1	24.9	48.5	42.6	8.9	51.2	40.8	7.9	7.0	37.6	55.4	53.2	39.0	7.8
ARIC-EA	35.0	47.8	17.2	6.3	36.1	57.5	24.1	49.9	26.0	7.4	39.1	53.5	49.4	41.8	8.8	54.9	38.3	6.8	7.6	40.1	52.3
ARIC-AA	56.3	39.2	4.5	10.4	44.6	45.0	13.6	47.3	39.2	5.2	40.8	53.9	7.2	37.5	55.3	52.4	40.4	7.2	18.5	50.6	30.9
Bogalusa Study	33.4	51.7	14.9	60.3	35.7	4.0	23.8	53.5	22.8	52.3	39.7	7.9	4.7	35.1	60.3	5.3	37.8	56.9	52.5	40.1	7.4
SiMES	33.3	49.9	16.8	NA	NA	NA	NA	NA	NA	31.7	31.7	20.3	46.6	42.8	10.6	8.7	39.9	51.4	53.0	39.0	8.0
SINDI	56.8	36.8	6.4	NA	NA	NA	NA	NA	NA	56.2	56.2	6.9	NA	NA	NA	2.0	21.1	76.9	56.5	36.6	6.9
SP2-SDCS-1M	20.8	49.8	29.4	NA	NA	NA	3.2	28.0	68.8	28.0	28.0	22.1	33.4	49.1	17.5	17.1	51.7	31.2	57.3	36.3	6.5
SP2-SDCS-610	22.1	48.9	29.1	NA	NA	NA	NA	NA	NA	27.8	27.8	22.6	38.7	46.7	14.7	18.2	48.5	33.3	55.1	38.3	6.7
SCES	NA	NA	NA	NA	NA	NA	NA	NA	NA	NA	NA	NA	NA	NA	NA	NA	NA	NA	NA	NA	NA
CHS	33.5	49.1	17.4	6.2	35.7	58.1	22.8	49.3	27.8	53.0	39.8	7.2	50.4	40.7	8.9	6.7	38.0	55.3	51.0	40.5	8.6
SCHS	28.2	49.9	21.9	NA	NA	NA	71.9	25.4	2.7	21.9	49.4	28.7	36.2	47.4	16.4	18.5	48.7	32.8	56.7	37.3	6.0

© 2019 BIRTH-GENE (BIG) Study Working Group. *JAMA Network Open.*

eTable 6. Associations between seven loci associated with birth weight and various anthropometric measures taken at birth (data from summary results)

			Birth weight (combined meta-analysis of European Discovery and Follow-up studies) [in grams]	
Locus	Index SNP	allele/Other allele	Beta (SE)	P-value
CCNL1	rs900400	C/T	−0.072 (0.006)	3.6E-38
ADCY5	rs9883204	C/T	−0.059 (0.006)	5.5E-20
HMGA2	rs1042725	T/C	−0.047 (0.005)	1.4E-19
CDKAL1	rs6931514	G/A	−0.050 (0.006)	1.5E-18
5q11.2	rs4432842	C/T	−0.034 (0.006)	0.000000046
LCORL	rs724577	C/A	−0.042 (0.006)	4.6E-11
ADRB1	rs1801253	G/C	−0.041 (0.007)	3.6E-09

Results are from inverse variance, fixed-effects meta-analysis of all available study samples of European ancestry. The effect allele for each SNP is labelled on the positive strand according to HapMap. The beta value is the change in trait z score per birth weight-lowering allele from linear regression, adjusted for sex and gestational age (where available), assuming an additive genetic model. To obtain the equivalent birth weight effect in grams, we multiplied by 484g, the median birth weight standard deviation of European studies in 2. There was little detectable heterogeneity between studies (all P > 0.01).
*Results are unadjusted for maternal genotype or birth length, but only in samples where maternal genotype or birth length is available (for direct comparison with the model that is adjusted for maternal genotype or birth length, respectively.)
The β value is the change in z score per birth weight–lowering allele from linear regression, adjusted for sex and gestational age (where available), assuming an additive genetic model. To obtain the equivalent birth weight effect in grams, we multiplied by 484 g, the median birth weight standard deviation of European studies.
Reference: EGG consortium, 2013, Nature Genetic

© 2019 BIRTH-GENE (BIG) Study Working Group. *JAMA Network Open.*

eTable 7. Genetic association of birth weight genetic variants with glycemic traits (data from summary results).

SNPs	Gene	Effect Alle	Other Alle	HbA1c, %			Fasting insulin, log (pmol/L)			T2DM			Fasting glucose, mmol/L			2h glucose, mmol/L		
				Beta	SE	P-value	Beta	SE	P-value	Beta	SE	P-value	Beta	SE	P-value	Beta	SE	P-value
rs1042725	HMGA2	C	T	-0.0005	0.0034	0.886	-0.0023	0.0038	0.537	-0.0392	0.0123	0.004	-0.0008	0.0036	0.819	-0.0200	0.0180	0.280
rs1801253	ADRB1	C	G	0.0002	0.0042	0.969	0.0025	0.0047	0.599	-0.0488	0.0168	0.003	-0.0056	0.0045	0.213	0.0019	0.0230	0.932
rs4432842	5q11.2	T	C	-0.0028	0.0037	0.453	0.0003	0.0042	0.940	0.0100	0.0126	0.560	-0.0070	0.0040	0.080	-0.0052	0.0200	0.799
rs6931514	CDKAL1	A	G	-0.0173	0.0038	0.000	0.0110	0.0042	0.010	-0.1484	0.0153	0.000	-0.0096	0.0041	0.019	-0.0420	0.0210	0.053
rs724577	LCORL	A	C	-0.0038	0.0038	0.319	-0.0089	0.0043	0.037	-0.0296	0.0148	0.027	-0.0074	0.0041	0.069	-0.0420	0.0200	0.041
rs900400	CCNL1	T	C	-0.0025	0.0035	0.485	-0.0052	0.0041	0.206	-0.0198	0.0149	0.210	-0.0035	0.0040	0.371	0.0320	0.0200	0.109
rs9883204	ADCY5	T	C	-0.0100	0.0041	0.014	0.0017	0.0047	0.721	-0.0953	0.0185	0.000	-0.0240	0.0045	0.000	-0.0970	0.0230	0.000

eTable 8. Sixty loci associated with birth weight ($P<5x10^{-8}$) in European ancestry and/or trans-ancestry (data from summary results).

Locus	SNP	Proxy SNP	Chr.	Alleles	EAF	European ancestry		Trans-ancestry	
				Effect/Other		β(SE) per SD	*P*-value	β(SE)	*P*-value
MTNR1B	rs10830963	rs10830963	11	G/C	0.27	0.023 (0.004)	2.9x10^{-8}	0.022 (0.004)	1.0x10^{-7}
APOLD1	rs11055034	rs11055034	12	C/A	0.73	0.022 (0.004)	1.8x10^{-7}	0.023 (0.004)	2.3x10^{-8}
SLC45A4	rs12543725	rs12543725	8	G/A	0.6	0.023 (0.004)	1.2x10^{-9}	0.022 (0.004)	1.9x10^{-9}
ITPR2	rs12823128	rs12823128	12	T/C	0.56	0.021 (0.004)	1.9x10^{-8}	0.020 (0.004)	3.2x10^{-8}
ANK1-NKX6-3	rs13266210	rs13266210	8	A/G	0.79	0.031 (0.005)	1.3x10^{-11}	0.030 (0.004)	1.6x10^{-11}
CCNL1-LEKR1	rs13322435	rs13322435	3	A/G	0.59	0.053 (0.004)	3.7x10^{-41}	0.052 (0.004)	1.3x10^{-42}
KREMEN1	rs134594	rs134594	22	C/T	0.35	0.023 (0.004)	1.0x10^{-8}	0.022 (0.004)	2.2x10^{-8}
HMGA2	rs1351394	rs1351394	12	T/C	0.48	0.044 (0.004)	1.9x10^{-32}	0.043 (0.004)	2.0x10^{-33}
EPAS1	rs1374204	rs1374204	2	T/C	0.7	0.047 (0.004)	6.2x10^{-29}	0.046 (0.004)	1.5x10^{-29}
L3MBTL3	rs1415701	rs1415701	6	G/A	0.73	0.025 (0.004)	2.6x10^{-9}	0.027 (0.004)	4.0x10^{-11}
LPAR1	rs2150052	rs2150052	9	T/A	0.5	0.021 (0.004)	2.2x10^{-8}	0.020 (0.004)	2.8x10^{-8}
NRIP1	rs2229742	rs2229742	21	G/C	0.87	0.036 (0.006)	2.2x10^{-9}	0.034 (0.006)	1.5x10^{-8}
PTH1R	rs2242116	rs2242116	3	A/G	0.39	0.022 (0.004)	1.4x10^{-8}	0.021 (0.004)	1.2x10^{-8}
LINC00332	rs2324499	rs2324499	13	G/C	0.67	0.022 (0.004)	7.3x10^{-8}	0.023 (0.004)	8.3x10^{-9}

© 2019 BIRTH-GENE (BIG) Study Working Group. *JAMA Network Open.*

PLEKHA1	rs2421016	rs2421016	10	T/C	0.48	0.021 (0.004)	$1.8x10^{-8}$	0.021 (0.004)	$6.1x10^{-9}$
ZBTB7B	rs3753639	rs3753639	1	C/T	0.23	0.031 (0.004)	$7.3x10^{-12}$	0.031 (0.004)	$1.3x10^{-12}$
MAFB	rs6016377	rs6016377	20	T/C	0.45	0.024 (0.004)	$9.5x10^{-10}$	0.024 (0.004)	$3.7x10^{-10}$
HHIP	rs6537307	rs6537307	4	G/A	0.48	0.025 (0.004)	$9.5x10^{-12}$	0.026 (0.004)	$1.3x10^{-12}$
TBX20	rs6959887	rs6959887	7	A/G	0.61	0.023 (0.004)	$1.5x10^{-9}$	0.021 (0.004)	$1.0x10^{-8}$
TRIB1	rs6989280	rs6989280	8	G/A	0.7	0.022 (0.004)	$2.2x10^{-7}$	0.022 (0.004)	$5.0x10^{-8}$
STRBP	rs700059	rs700059	9	G/A	0.16	0.033 (0.005)	$4.7x10^{-10}$	0.036 (0.005)	$1.2x10^{-12}$
ADRB1	rs7076938	rs7076938	10	T/C	0.73	0.036 (0.004)	$4.7x10^{-18}$	0.035 (0.004)	$4.7x10^{-18}$
HMGA1	rs7742369	rs7742369	6	G/A	0.19	0.028 (0.005)	$1.0x10^{-8}$	0.027 (0.005)	$1.1x10^{-8}$
IGF1	rs7964361	rs7964361	12	A/G	0.08	0.039 (0.007)	$4.7x10^{-9}$	0.038 (0.007)	$9.7x10^{-9}$
GNA12	rs798489	rs798489	7	C/T	0.74	0.023 (0.004)	$2.0x10^{-8}$	0.024 (0.004)	$5.0x10^{-9}$
5q11.2	rs854037	rs854037	5	A/G	0.8	0.027 (0.005)	$2.2x10^{-8}$	0.025 (0.005)	$3.5x10^{-8}$
LCORL	rs925098	rs925098	4	G/A	0.28	0.034 (0.004)	$5.4x10^{-16}$	0.032 (0.004)	$1.3x10^{-15}$
CPA3	rs10935733	rs10935733	3	T/C	0.42	0.022 (0.004)	$9.2x10^{-9}$	0.023 (0.004)	$6.2x10^{-10}$
PLAC1	rs11096402	rs11096402	X	G/A	0.25	0.028 (0.005)	$1.3x10^{-9}$	N/A	N/A
IGF2BP3	rs11765649	rs11765649	7	T/C	0.76	0.027 (0.004)	$5.8x10^{-10}$	0.026 (0.004)	$1.0x10^{-9}$
SP6-SP2	rs12942207	rs12942207	17	C/T	0.3	0.022 (0.004)	$5.1x10^{-8}$	0.024 (0.004)	$3.0x10^{-9}$
YKT6-GCK	rs138715366	rs138715366	7	C/T	0.99	0.241 (0.023)	$7.2x10^{-26}$	0.244 (0.023)	$1.4x10^{-26}$
ABCC9	rs139975827	rs139975827	12	G/A	0.63	0.025 (0.004)	$1.1x10^{-8}$	0.022 (0.004)	$1.0x10^{-7}$
SUZ12P1-CRLF3	rs144843919	rs144843919	17	G/A	0.96	0.066 (0.012)	$1.4x10^{-8}$	0.068 (0.011)	$1.5x10^{-9}$
RNF219-AS1	rs1819436	rs1819436	13	C/T	0.87	0.033 (0.006)	$6.3x10^{-9}$	0.033 (0.005)	$1.8x10^{-9}$
RB1	rs2854355	rs2854355	13	G/A	0.26	0.023 (0.004)	$9.8x10^{-8}$	0.024 (0.004)	$2.2x10^{-8}$
DTL	rs61830764	rs61830764	1	A/G	0.36	0.022 (0.004)	$5.6x10^{-8}$	0.022 (0.004)	$4.5x10^{-8}$
SREBF2	rs62240962	rs62240962	22	C/T	0.92	0.047 (0.007)	$9.7x10^{-12}$	0.047 (0.007)	$3.7x10^{-2}$
MLXIPL	rs62466330	rs62466330	7	C/T	0.07	0.049 (0.008)	$1.2x10^{-12}$	0.051 (0.007)	$5.9x10^{-12}$
FCGR2B	rs72480273	rs72480273	1	C/A	0.17	0.031 (0.005)	$8.0x10^{-10}$	0.030 (0.005)	$1.5x10^{-9}$
INS-IGF2	rs72851023	rs72851023	11	T/C	0.07	0.048 (0.008)	$2.9x10^{-10}$	0.046 (0.007)	$6.8x10^{-10}$
ATAD2B	rs7575873	rs7575873	2	A/G	0.88	0.038 (0.006)	$1.3x10^{-11}$	0.036 (0.006)	$6.2x10^{-11}$
EBF1	rs7729301	rs7729301	5	A/G	0.72	0.024 (0.004)	$1.6x10^{-8}$	0.025 (0.004)	$1.3x10^{-9}$
HIST1H2BE	rs9379832	rs9379832	6	A/G	0.71	0.023 (0.004)	$6.6x10^{-8}$	0.024 (0.004)	$1.2x10^{-8}$
NT5C2	rs74233809	rs11191582	10	C/T	0.08	0.037 (0.007)	$5.2x10^{-8}$	0.039 (0.006)	$1.8x10^{-9}$

© 2019 BIRTH-GENE (BIG) Study Working Group. *JAMA Network Open.*

PEPD	rs10402712	rs11667352	19	A/G	0.27	0.022 (0.004)	$4.4x10^{-7}$	0.023 (0.004)	$2.3x10^{-8}$
ACTL9	rs61154119	rs11670067	19	T/G	0.84	0.028 (0.005)	$1.1x10^{-7}$	0.028 (0.005)	$2.3x10^{-8}$
ADCY5	rs11719201	rs11708067	3	T/C	0.23	0.046 (0.004)	$2.4x10^{-26}$	0.046 (0.004)	$6.4x10^{-27}$
FES	rs12906125	rs1894400	15	G/A	0.69	0.023 (0.004)	$1.7x10^{-8}$	0.023 (0.004)	$1.0x10^{-8}$
IGF1R	rs7402982	rs2017500	15	A/G	0.42	0.023 (0.004)	$2.3x10^{-9}$	0.023 (0.004)	$1.1x10^{-9}$
JAG1	rs6040076	rs2206815	20	C/G	0.51	0.023 (0.004)	$2.0x10^{-9}$	0.022 (0.004)	$7.2x10^{-9}$
CLDN7	rs113086489	rs222857	17	T/C	0.55	0.031 (0.004)	$9.1 x10^{-16}$	0.030 (0.004)	$1.3X10^{-15}$
HHEX-IDE	rs61862780	rs2497306	10	T/C	0.52	0.028 (0.004)	$3.0x10^{-14}$	0.028 (0.004)	$9.5x10^{-15}$
WNT4-ZBTB40	rs2473248	rs2744728	1	C/T	0.87	0.033 (0.006)	$1.1x10^{-8}$	0.033 (0.005)	$1.1x10^{-9}$
GPR139	rs1011939	rs2764742	16	G/A	0.31	0.022 (0.004)	$1.3x10^{-7}$	0.024 (0.004)	$2.7x10^{-9}$
PTCH1	rs28510415	rs28446116	9	G/A	0.09	0.056 (0.007)	$1.5x10^{-17}$	0.053 (0.006)	$4.0x10^{-16}$
PHF19	rs7847628	rs3933326	9	G/A	0.67	0.023 (0.004)	$1.0x10^{-8}$	0.023 (0.004)	$5.4x10^{-9}$
C20orf203	rs28530618	rs6057610	20	A/G	0.5	0.026 (0.004)	$7.7x10^{-12}$	0.024 (0.004)	$8.4x10^{-11}$
CDKAL1	rs35261542	rs7756992	6	C/A	0.73	0.044 (0.004)	$4.4x10^{-27}$	0.044 (0.004)	$9.7x10^{-29}$
ESR1	rs1101081	rs851978	6	C/T	0.73	0.038 (0.004)	$1.6x10^{-19}$	0.037 (0.004)	$6.1 x10^{-20}$

Sixty loci associated with birth weight ($P < 5 x 10^{-8}$) in European ancestry meta-analysis of up to 143,677 individuals and/or trans-ancestry meta-analysis of up to 153,781 individuals.
BW was z-score transformed separately in males and females after excluding non-singletons and premature births and adjusting for gestational age where available.
a, Effects (beta values) are aligned to the BW-raising allele.
EAF was obtained from the trans-ancestry meta-analysis, except for PLAC1, for which the EAF was obtained from the European ancestry meta-analysis due to lack of X chromosome data from the non-European studies.
Chr., chromosome; bp, base pair; EAF, effect allele frequency; SE, standard error.
The lead SNP (or proxy, EUR r2>0.6) at the 60 BW loci was queried in publicly available GWAS meta-analysis datasets or in GWAS result obtained through collaboration79. Results were available for 53 of those loci and the extracted z-score (allelic effect/SE) was aligned to the BW-raising allele.

© 2019 BIRTH-GENE (BIG) Study Working Group. *JAMA Network Open.*

eTable 9. Genetic association of birth weight related 60 genetic variants with glycemic traits (data from summary results).

Genes	Index SNPs	Proxy SNPs	Chr.	Effect/Other	EAF	BW Europeans			BW Trans-ethnic results			Fasting glucose, mmol/L			Fasting insulin, log (pmol/L)			HbA1C1 , %			2hGlu, mmol/L			T2DM		
						Beta	SE	P-value	Beta	SE	P-value	Beta	SE	P-value	Beta	SE	P-value	Beta	SE	P-value	Beta	SE	P-value	Beta	SE	P-value
MTNR1B	rs10830963	rs10830963	11	G/C	0.27	0.023	0.004	2.9x10-8	0.022	0.004	1.0x10-7	0.079	0.0045	1.26E-68	-0.004	0.0047	0.3988	0.0238	0.004	2.99E-09	0.056	0.022	0.01172	0.0374265	0.0071502	2.00E-07
APOLD1	rs11055034	rs11055034	12	C/A	0.73	0.022	0.004	1.8x10-7	0.023	0.004	2.3x10-8	-0.0011	0.0043	0.7883	-0.0025	0.0045	0.5677	0.0006	0.0039	0.881	0.022	0.022	0.3057	-0.0086002	0.0076437	0.34
SLC45A4	rs12543725	rs12543725	8	G/A	0.6	0.023	0.004	1.2x10-9	0.022	0.004	1.9x10-9	-0.0018	0.0039	0.6512	0.0037	0.004	0.3533	-0.0008	0.0036	0.8162	0.013	0.019	0.4972	0.0086002	0.0065189	0.23
ITPR2	rs12823128	rs12823128	12	T/C	0.56	0.021	0.004	1.9x10-8	0.02	0.004	3.2x10-8	-0.0003	0.0036	0.942	-0.0009	0.0038	0.8026	0.0023	0.0034	0.504	-0.018	0.018	0.3339	-0.0043214	0.0065835	0.38
ANK1-NKX6-3	rs13266210	rs13266210	8	A/G	0.79	0.031	0.005	1.3x10-11	0.03	0.004	1.6x10-11	0.0075	0.0046	0.09988	-0.0082	0.0047	0.08401	0.0128	0.0042	0.002336	0.04	0.023	0.09011	0.0293838	0.0082872	1.00E-04
CCNL1-LEKR1	rs13322435	rs13322435	3	A/G	0.59	0.053	0.004	3.7x10-41	0.052	0.004	1.3x10-42	-0.0043	0.0041	0.2996	-0.0049	0.0043	0.2477	-0.0022	0.0037	0.5489	0.026	0.021	0.2222	-0.0128372	0.0064301	0.086
KREMEN1	rs134594	rs134594	22	C/T	0.35	0.023	0.004	1.0x10-8	0.022	0.004	2.2x10-8	0.0009	0.0038	0.8162	-0.0047	0.004	0.2383	-0.0004	0.0036	0.9107	-0.0016	0.019	0.9352	-0.0128372	0.0064301	0.021
HMGA2	rs1351394	rs1351394	12	T/C	0.48	0.044	0.004	1.9x10-32	0.043	0.004	2.0x10-33	-0.0004	0.0037	0.9053	-0.0045	0.0038	0.2313	0.001	0.0034	0.7578	-0.027	0.018	0.1482	-0.0128372	0.0064301	0.044
EPAS1	rs1374204	rs1374204	2	T/C	0.7	0.047	0.004	6.2x10-29	0.046	0.004	1.5x10-29	-0.0071	0.0047	0.1275	-0.0053	0.0049	0.2745	0.0036	0.0044	0.4077	-0.014	0.024	0.5563	0.0128372	0.0064301	0.065
L3MBTL3	rs1415701	rs1415701	6	G/A	0.73	0.025	0.004	2.6x10-9	0.027	0.004	4.0x10-11	0.0076	0.0043	0.07685	-0.006	0.0045	0.1831	0.0019	0.0038	0.6155	-0.021	0.023	0.3466	-0.0128372	0.0064301	0.026
LPAR1	rs2150052	rs2150052	9	T/A	0.5	0.021	0.004	2.2x10-8	0.02	0.004	2.8x10-8	-0.003	0.0036	0.413	-0.0017	0.0038	0.6558	0.0032	0.0034	0.3466	0.018	0.018	0.33	-0.0043214	0.0054587	0.43
NRIP1	rs2229742	rs2229742	21	G/C	0.87	0.036	0.006	2.2x10-9	0.034	0.006	1.5x10-8	0.0045	0.0063	0.4724	0.0052	0.0066	0.4285	0.0007	0.0058	0.9039	-0.0059	0.031	0.8514	-0.0043214	0.0131786	0.73
PTH1R	rs2242116	rs2242116	3	A/G	0.39	0.022	0.004	1.4x10-8	0.021	0.004	1.2x10-8	0.0072	0.004	0.0686	0.0006	0.0041	0.8897	-0.0084	0.0037	0.02313	0.015	0.02	0.4411	-0.0170333	0.0053532	0.014
LINC00332	rs2324499	rs2324499	13	G/C	0.67	0.022	0.004	7.3x10-8	0.023	0.004	8.3x10-9	-0.0057	0.0045	0.1987	0.0028	0.0047	0.5568	-0.0108	0.004	0.007618	-0.045	0.023	0.05147	-0.0086002	0.0086938	0.29
PLEKHA1	rs2421016	rs2421016	10	T/C	0.48	0.021	0.004	1.8x10-8	0.021	0.004	6.1x10-9	0.0032	0.0037	0.3787	-0.0015	0.0038	0.6857	0.0025	0.0034	0.4565	-0.025	0.019	0.1813	-0.0211893	0.0052517	1.90E-05
MAFB	rs6016377	rs6016377	20	T/C	0.45	0.024	0.004	9.5x10-10	0.024	0.004	3.7x10-10	0.008	0.004	0.04602	-0.0091	0.0042	0.03002	0.0036	0.0036	0.3165	-0.0069	0.02	0.731	0.0086002	0.0065189	0.18
HHIP	rs6537307	rs6537307	4	G/A	0.48	0.025	0.004	9.5x10-12	0.026	0.004	1.3x10-12	0.0059	0.0037	0.1104	-0.002	0.0038	0.6088	-0.0099	0.0035	0.004119	-0.019	0.019	0.3188	-0.0043214	0.005513	0.69
TBX20	rs6959887	rs6959887	7	A/G	0.61	0.023	0.004	1.5x10-9	0.021	0.004	1.0x10-8	0.003	0.0037	0.4111	0.0028	0.0039	0.4613	-0.0004	0.0035	0.9115	0.012	0.019	0.5378	-0.0043214	0.005513	0.64
TRIB1	rs6989280	rs6989280	8	G/A	0.7	0.022	0.004	2.2x10-7	0.022	0.004	5.0x10-8	0.0054	0.0046	0.2387	0.0005	0.0047	0.9064	0.0018	0.004	0.6589	-0.035	0.023	0.1211	0.0043214	0.0065835	0.68
STRBP	rs700059	rs700059	9	G/A	0.16	0.033	0.005	4.7x10-10	0.036	0.005	1.2x10-12	-0.0051	0.0052	0.326	-0.01	0.0054	0.05566	-0.0068	0.005	0.178	-0.032	0.026	0.2228	-0.0253059	0.0062728	2.00E-04
ADRB1	rs7076938	rs7076938	10	T/C	0.73	0.036	0.004	4.7x10-18	0.035	0.004	4.7x10-18	-0.0054	0.0045	0.2303	0.0026	0.0046	0.5753	0.0007	0.0042	0.8738	0.0025	0.023	0.9124	-0.0170333	0.0063935	0.0068
HMGA1	rs7742369	rs7742369	6	G/A	0.19	0.028	0.005	1.0x10-8	0.027	0.005	1.1x10-8	-0.002	0.0053	0.7053	0.0082	0.0056	0.1404	-0.0064	0.0052	0.2194	-0.042	0.029	0.1436	-0.0043214	0.00878	0.63
IGF1	rs7964361	rs7964361	12	A/G	0.08	0.039	0.007	4.7x10-9	0.038	0.007	9.7x10-9	0.0061	0.0066	0.36	0.0012	0.0069	0.8614	0.0089	0.0063	0.1571	-0.031	0.034	0.3573	-0.0253059	0.0114532	0.015

© 2019 BIRTH-GENE (BIG) Study Working Group. *JAMA Network Open.*

GNA12	rs798489	rs798489	7	C/T	0.74	0.023	0.004	2.0x10-8	0.024	0.004	5.0x10-9	0.0004	0.0041	0.9188	0.0016	0.0043	0.7156	0.0034	0.0038	0.3707	0.0009	0.02	0.9639	0.00001	0.0077198	0.82
5q11.2	rs854037	rs854037	5	A/G	0.8	0.027	0.005	2.2x10-8	0.025	0.005	3.5x10-8	-0.0092	0.0047	0.05255	0.0025	0.005	0.6186	-0.0007	0.0044	0.8736	0.02	0.024	0.4014	0.0128372	0.0064301	0.042
LCORL	rs925098	rs925098	4	G/A	0.28	0.034	0.004	5.4x10-16	0.032	0.004	1.3x10-15	-0.0064	0.0041	0.1155	-0.0082	0.0042	0.05414	-0.0044	0.0039	0.2498	-0.043	0.021	0.0365	-0.0086002	0.0065189	0.11
ZBTB7B	rs3753639	rs3753639	1	C/T	0.23	0.031	0.004	7.3x10-12	0.031	0.004	1.3x10-12	NA	NA	NA	NA	NA	NA	NA	NA	NA	NA	NA	NA	-0.0086002	0.0227782	0.72
NT5C2	rs74233809	rs11191582	10	C/T	0.08	0.037	0.007	5.2x10-8	0.039	0.006	1.8x10-9	0.0002	0.0064	0.9756	-0.0015	0.0066	0.8145	NA	NA	NA	NA	NA	NA	0.0128372	0.0074933	0.075
PEPD	rs10402712	rs11667352	19	A/G	0.27	0.022	0.004	4.4x10-7	0.023	0.004	2.3x10-8	-0.0059	0.0038	0.1234	-0.01	0.004	0.008841	-0.0045	0.006	0.4497	-0.036	0.033	0.2727	-0.0086002	0.0065189	0.16
ACTL9	rs61154119	rs11670067	19	T/G	0.84	0.028	0.005	1.1x10-7	0.028	0.005	2.3x10-8	-0.0037	0.0053	0.4837	-0.0091	0.0054	0.0923	-0.0016	0.0035	0.6429	-0.015	0.019	0.4157	0.0043214	0.00878	0.66
ADCY5	rs11719201	rs11708067	3	T/C	0.23	0.046	0.004	2.4x10-26	0.046	0.004	6.4x10-27	-0.027	0.0047	8.72E-09	0.0031	0.0049	0.5302	-0.0121	0.0054	0.0236	-0.02	0.031	0.5228	-0.0413927	0.0070207	1.40E-08
IGF1R	rs7402982	rs2017500	15	A/G	0.42	0.023	0.004	2.3x10-9	0.023	0.004	1.1x10-9	-0.0065	0.007	0.354	-0.0027	0.0068	0.6883	-0.0145	0.0042	0.0005046	-0.093	0.023	6.642E-05	-0.0128372	0.0107647	0.23
JAG1	rs6040076	rs2206815	20	C/G	0.51	0.023	0.004	2.0x10-9	0.022	0.004	7.2x10-9	-0.0014	0.0038	0.7109	0.0017	0.0039	0.6728	0.01	0.0059	0.0875	NA	NA	NA	0.0001	0.0066494	0.99
CLDN7	rs113086489	rs222857	17	T/C	0.55	0.031	0.004	9.1 x10-16	0.03	0.004	1.3X10-15	-0.0075	0.0041	0.06633	0.0037	0.0042	0.3886	-0.0068	0.0035	0.05263	0.016	0.019	0.4039	-0.0086002	0.0065189	0.15
HHEX-IDE	rs61862780	rs2497306	10	T/C	0.52	0.028	0.004	3.0x10-14	0.028	0.004	9.5x10-15	-0.0066	0.0036	0.06918	0.0036	0.0038	0.3373	-0.0005	0.0037	0.8974	0.04	0.021	0.04996	-0.049218	0.0059366	7.70E-14
WNT4-ZBTB40	rs2473248	rs2744728	1	C/T	0.87	0.033	0.006	1.1x10-8	0.033	0.005	1.1x10-9	-0.0018	0.0055	0.7387	0.0036	0.0056	0.5255	-0.0044	0.0034	0.2048	-0.021	0.019	0.2581	0.0043214	0.0077198	0.67
GPR139	rs1011939	rs2764742	16	G/A	0.31	0.022	0.004	1.3x10-7	0.024	0.004	2.7x10-9	-0.0004	0.0039	0.9268	0.0031	0.004	0.4388	-0.0098	0.0051	0.05208	-0.0094	0.028	0.7351	-0.0043214	0.005513	0.61
PHF19	rs7847628	rs3933326	9	G/A	0.67	0.023	0.004	1.0x10-8	0.023	0.004	5.4x10-9	0.0048	0.0042	0.2491	0.0001	0.0044	0.9787	-0.005	0.0037	0.171	0.011	0.02	0.5924	-0.0001	0.0066494	0.99
C20orf203	rs28530618	rs6057610	20	A/G	0.5	0.026	0.004	7.7x10-12	0.024	0.004	8.4x10-11	-0.0028	0.0037	0.4517	0.0054	0.0039	0.161	-0.0016	0.004	0.6848	0.0024	0.021	0.9098	-0.0001	0.005513	0.87
CDKAL1	rs35261542	rs7756992	6	C/A	0.73	0.044	0.004	4.4x10-27	0.044	0.004	9.7x10-29	-0.0095	0.0041	0.02038	0.011	0.0042	0.01073	0.002	0.0034	0.5627	-0.013	0.018	0.4656	-0.0606978	0.0057816	1.60E-26
ESR1	rs1101081	rs851978	6	C/T	0.73	0.038	0.004	1.6x10-19	0.037	0.004	6.1 x10-20	0.0006	0.0041	0.8855	0.0011	0.0042	0.7921	-0.0178	0.0038	3.204E-06	-0.045	0.021	0.03732	-0.0043214	0.0065835	0.52
FES	rs12906125	rs1894400	15	G/A	0.69	0.023	0.004	1.7x10-8	0.023	0.004	1.0x10-8	NA	NA	NA	NA	NA	NA	0.0002	0.0038	0.9567	-0.0068	0.021	0.7513	0.0086002	0.0162421	0.61
PTCH1	rs28510415	rs28446116	9	G/A	0.09	0.056	0.007	1.5x10-17	0.053	0.006	4.0x10-16	NA	NA	NA	NA	NA	NA	-0.0089	0.008	0.2705	NA	NA	NA	-0.0211893	0.0303565	0.47

© 2019 BIRTH-GENE (BIG) Study Working Group. *JAMA Network Open.*

eTable 10. Association of the genetic risk score with birth weight and F statistic for the instrumental variable in the NHS, HPFS, and WHI cohorts

Study	F statistic test for Instrumental variable			
	Beta	SE	P-value	F statistics
NHS	0.015	0.0035	<.0001	18.28
HPFS	0.026	0.006	<.0001	18.34

Linear regression models were used to examine the association of genetic risk score with birth weight.

© 2019 BIRTH-GENE (BIG) Study Working Group. *JAMA Network Open.*

eTable 11. Association of birth weight genetic risk score with confounders according to quartiles of the GRS in the NHS, HPFS and WHI studies.

Confounders	NHS					HPFS				
	Q1	Q2	Q3	Q4	P-value	Q1	Q2	Q3	Q4	P-value
n	2675	2273	3001	2724		2675	2273	3001	2724	
Age (years)	54 ± 6	54 ± 6	54 ± 6	54 ± 6	0.61	54 ± 6	54 ± 6	54 ± 6	54 ± 6	0.61
BMI (kg/m2)	25.8 ± 4.9	25.7 ± 4.6	25.8 ± 4.7	25.8 ± 4.8	0.67	25.8 ± 4.9	25.7 ± 4.6	25.8 ± 4.7	25.8 ± 4.8	0.67
Total energy intake (MJ/day)	7.46 ± 2.03	7.50 ± 2.01	7.47 ± 2.05	7.44 ± 2.02	0.74	7.46 ± 2.03	7.50 ± 2.01	7.47 ± 2.05	7.44 ± 2.02	0.74
Alcohol (g/day)	6.4 ± 10.2	6.6 ± 10.7	6.4 ± 10.1	6.2 ± 9.8	0.61	6.4 ± 10.2	6.6 ± 10.7	6.4 ± 10.1	6.2 ± 9.8	0.61
Current smokers (%)	17.6	14.5	15.2	16.1	0.18	17.6	14.5	15.2	16.1	0.18

Continued

Variant	Chr	Position	Eff. allele	Body mass index (BMI)		Alcohol intake frequency.		Smoking status	
				beta	pv	beta	pv	beta	pv
rs1042725	12	66358347	C	0.006785	0.39569	-0.01309	0.31209	-0.00136	0.28965
rs1801253	10	1.16E+08	C	-0.00686	0.451	0.001565	0.62774	-0.00107	0.46301
rs4432842	5	57172078	C	-0.01078	0.21557	0.003539	0.25052	-0.00337	0.015596
rs6931514	6	20703952	G	-0.03898	1.81E-05	-0.00253	0.43045	0.001758	0.22677
rs724577	4	17993410	C	0.010971	0.22786	0.003817	0.23647	-0.00189	0.19634
rs900400	3	1.57E+08	T	0.029383	0.000374	-0.002	0.49269	-0.00106	0.42387
rs9883204	3	1.23E+08	C	-0.02573	0.004625	-0.00633	0.048369	0.002168	0.13584

CData are mean ± SD or percentage (%).
p values for difference are based on Analysis of variance (ANOVA) for continuous data or chi square test for categorical data

© 2019 BIRTH-GENE (BIG) Study Working Group. *JAMA Network Open.*

eAppendix: Description of included studies

NHS

The NHS began in 1976, when 121,700 female registered nurses aged 30-55 y residing in 11 states were recruited to complete a baseline questionnaire about their lifestyle and medical history. Questionnaires were collected at baseline and biennially thereafter, to update information on lifestyle factors and the occurrence of chronic diseases. In the current analysis, we used 1990 as baseline in the NHS, when the earliest complete dietary data were collected. Our analysis included 13,000 women whose genotype data were available. All of the participants were Caucasians and were free of cancer at baseline. The study protocol was approved by the institutional review boards of Brigham and Women's Hospital and Harvard School of Public Health.

HPFS

The HPFS was initiated in 1986, and was composed of 51,529 male dentists, pharmacists, veterinarians, optometrists, osteopathic physicians, and podiatrists, aged 40-75 y at baseline. The male participants returned a baseline questionnaire about detailed medical history, lifestyle, and usual diet. Questionnaires were collected at baseline and biennially thereafter, to update information on lifestyle factors and the occurrence of chronic diseases. In the current analysis, we used 1990 as baseline in the HPFS, when the earliest complete dietary data were collected. Our analysis included 8,000 men whose genotype data were available. All of the participants were Caucasians and were free of cancer at baseline. The study protocol was approved by the institutional review boards of Brigham and Women's Hospital and Harvard School of Public Health.

DCH

The present study includes a case-cohort sample of 1,812 cases who developed T2DM before 31 Dec 2006, and 1,633 randomly selected controls, nested within the population-based Diet, Cancer and Health cohort. Diet, Cancer and Health is a Danish prospective cohort study originally aimed at investigating the associations between dietary habits, lifestyle, and cancer development. The participants were recruited during 1993-1997. A total of about 160,725 individuals were invited by mail, and 57,053 were enrolled into the study cohort. The participants are men and women born in Denmark, living in the greater Copenhagen or Aarhus areas, aged 50-64 years, and with no previous cancer diagnosis.

GOYA

The GOYA (Male) cohort is a longitudinal case-cohort (obese, non-obese) study comprising a randomly (1%) selected control group and all extremely overweight men identified among 362,200 Caucasian men examined at the mean age of 20 years at the draft boards in Copenhagen and its surrounding areas during 1943–1977. Obesity was defined as 35% overweight relative to a local standard in use at the time (mid 1970's), corresponding to a BMI ⩾31.0 kg/m2, which proved to be above the 99th percentile. All of the obese and 50% of the random sampled controls, who were still living in the region, were invited to a follow-up survey in 1992–94 at the mean age of 46 years, at which time the blood samples were taken and genotyping were performed for a total of 673 extremely overweight and 792 controls. With a sampling fraction of 0.5% (50% of 1%), the controls represent about 158,000 men among whom the case group was the most obese.

Raine Study

The Western Australian Pregnancy Cohort (Raine) Study is a prospective pregnancy cohort where 2,900 mothers where recruited between 1989 and 1991. Recruitment took place at Western Australia's major perinatal centre, King Edward Memorial Hospital, and nearby private practices. Women who had sufficient English language skills, an expectation to deliver at King Edward Memorial Hospital, and an intention to reside in Western Australia to allow for future follow-up of their child were eligible for the study. The Raine Study is known to be one of the largest successfully prospective cohorts richly phenotyped at multiple time points over pregnancy, infancy, childhood adolescence, and young adult. The mothers completed questionnaires regarding their children and the children had physical examinations at ages 1, 2, 3, 6, 8, 10, 14, 17, 20 and 22 years.

© 2019 BIRTH-GENE (BIG) Study Working Group. *JAMA Network Open.*

COPSAC REGISTRY

The COPSAC Registry is part of the Danish birth register comprising childhood asthma cases with data available on birth related traits plus genomic profiling. This cohort contributes in the birthweight-gene variant associations necessary for the current study.

COPSAC-CHILDREN

The COPSAC cohort comprises birth weight measures and data combined from two Danish birth cohorts: the COPSAC2000 and the COPSAC2010 studies. The COPSAC2000 cohort is a prospective clinical birth cohort study of 411 children of asthmatic mothers whereas the COPSAC2010 birth cohort is a population based longitudinal clinical study of 700 pregnant women and their offspring.

GESUS

The Danish General Suburban Population Study Invited all inhabitants ≥ 30 years old and 25% of people 20-29 years old with a valid Danish civil registration number from 2010-2013. 21,205 adults participated (participation rate 43%). Data come from questionnaires and clinical examinations. For this study we included 14,169 adults who were term at birth (871 were preterm, the rest unknown) and with a valid birth weight. 99% of participants were white.

Generation R

The Generation R Study is a population-based prospective cohort study from fetal life onwards in Rotterdam, the Netherlands. All children were born between April 2002 and January 2006. Enrolmentwas aimed at early pregnancy, but was allowed until birth of the child. A total of 9,778 mothers and their children were included. The current analysis includes the 2,701 European-ancestry children with genome-wide scan data and birth weight data.The study was approved by the Medical Ethics Committee of the Erasmus MC, University Medical Center Rotterdam and written informed consent was obtained for all participants.

YFS

The Cardiovascular Risk in Young Finns (YFS) is a population-based 27 year follow up-study (http://med.utu.fi/cardio/youngfinnsstudy/). The first cross-sectional survey was conducted in 1980, when 3,596 Caucasian subjects aged 3-18 years participated. In adulthood, the latest 27-year follow-up study was conducted in 2007 (ages 30-45 years) with 2,204 participants. The study cohort for the present analysis comprised subjects who had participated in the study in 2007 and had validated dietary data from FFQ, available genotype and other risk factor data. The dietary intake of nutrients was assessed using a modified 131-item food frequency questionnaire developed by the Finnish National Institute for Health and Welfare. The study was approved by the local Ethical Committees and was performed according to Helsinki declaration.

MESA

The Multi-Ethnic Study of Atherosclerosis (MESA) is a study of the characteristics of subclinical cardiovascular disease (disease detected non-invasively before it has produced clinical signs and symptoms) and the risk factors that predict progression to clinically overt cardiovascular disease or progression of the subclinical disease. MESA researchers study a diverse, population-based sample of 6,814 asymptomatic men and women aged 45-84 at baseline. Thirty-eight percent of the recruited participants were white, 28 percent African-American, 22 percent Hispanic, and 12 percent Asian, predominantly of Chinese descent. Participants were recruited from six field centers across the United States and followed-up five times with an average time period of follow-up of 2 years between each visit. Data from four visits (exam1 to exam5) was used for the analysis. The tenets of the Declaration of Helsinki were followed and institutional review board approval was granted at all MESA sites. Written informed consent was obtained from each participant.

MDC

The Malmö Diet and Cancer study is a population-based cohort with 30,446 adults (62% women; 45-73 years) recruited at baseline in 1991-1996. In a cardiovascular sub-cohort 6103 adults were randomly selected from the parent Malmö cohort. 5040 adults with genotype information and who provided valid dietary information were eligible for the current analysis.

© 2019 BIRTH-GENE (BIG) Study Working Group. *JAMA Network Open.*

WGHS

The WGHS is a prospective cohort of initially healthy U.S. women. Study participants were health professionals who were age 45 years and older and free of major chronic disease including cancer and cardiovascular disease at study entry (1992-1995). A total of 23,294 had confirmed self-reported European ancestry and genotyping information. There were a total of 12,768 WGHS participants who did not qualify for one or more of the following conditions: were a preterm birth, one of a multiple birth, had missing information for either of these variables, or had diabetes or hypertension at baseline. The present study was approved by the institutional review boards of Brigham and Women's Hospital and Harvard School of Public Health (Boston, MA)

THISEAS

The Hellenic Study of Interactions between SNPs and Eating in Atherosclerosis Susceptibility (THISEAS) study is a case- control study designed to investigate the association between genetic and lifestyle environmental factors and the risk of coronary artery disease in men and women aged >25 yrs. The control group consists of individuals with no history of cardiovascular disease, while cases are individuals with coronary artery disease. Hematological, biochemical and anthropometric measurements were conducted to all participants. Dietary assessment and physical activity data were collected through face-to-face interview by well-trained scientists. Metabochip was used for DNA analysis. Exclusion criteria for the control group were history of cardiovascular disease, cancer and/ or other inflammatory disease.

DGDG

T2DM case-control study including 1,376 individuals coming from 3 French cohorts: 679 diabetic subjects recruited at the laboratory CNRS UMR8199 and at the Endocrinology-Diabetology Department of the Corbeil-Essonnes Hospital, 697 controls coming from the cohort DESIR (Epidemiological Study on the Insulin Resistance syndrome). The inclusion criteria for cases are: T2DM mellitus 1997 American Diabetes Association (ADA) definition, family history of diabetes in first degree relatives, Body Mass Index (BMI) < 30 kg/m². Controls are at least 45 years old, with a normal fasting glucose according to 1997 ADA and a BMI < 27 kg/m². All individuals were genotyped with the Illumina HAP300 chip. Participants laying outside from CEPH cluster, defined by HapMap were discarded. Informed consent was obtained from all participants, and the French ethics committee approved the study protocol.

PREDIMED-Valencia

The PREDIMED-Valencia study was initiated in 2003 including 1,094 participants. Valencia is one of the field centers participating in the PREvencion con DietaMEDiterranea (PREDIMED) trial. Eligible participants were community-dwelling persons (55-80 years for men; 60-80 years for women) who fulfilled at least one of two criteria: type2 diabetes or 3 or more cardiovascular risk factors. Here we used an observational cohort design and we included 1,023 PREDIMED-Valencia participants with valid genotype data for the analyzed SNPs. Prevalence of T2D was analyzed at baseline. Validated FFQ questionnaires were used at baseline. Weight and BMI were directly measured. The Institutional Review Board of the University of Valencia approved the study protocol.

LIFE Leipzig Heart

LIFE Leipzig Heart is an observational of patients with suspect or confirmed coronary artery disease (PMID 22216169). Recruitment comprised patients receiving coronary angiography for diagnostic reasons for the first time, patients with confirmed stable left main coronary artery disease and patients with acute myocardial infarction. The study meets the ethical standards of the Declaration of Helsinki. It has been approved by the Ethics Committee of the Medical Faculty of the University of Leipzig, Germany (Reg. No 276-2005) and is registered at ClinicalTrials.gov (NCT00497887). Written informed consent including agreement with genetic analyses was obtained from all participants.

LIFE-Adult

© 2019 BIRTH-GENE (BIG) Study Working Group. *JAMA Network Open.*

LIFE-Adult is a cohort of about 10,000 inhabitants of the city of Leipzig (Saxony, Germany) with primary age range between 40 and 79 years. Participants were phenotyped for several health and disease related parameters, including blood sampling (see PMID 26197779 for further details). All subjects gave written informed consent to participate in the examinations. The procedures were conducted according to the Declaration of Helsinki and were approved by the University of Leipzig's ethics committee (registration-number: 263-2009-14122009). LIFE-Adult is part of the Leipzig Research Center for Civilisation Diseases (LIFE).

HELENA

Participants were recruited from 2006 to 2007 in 10 centres from 9 European countries (Athens and Heraklion in Greece, Dortmund in Germany, Ghent in Belgium, Lille in France, Pècs in Hungary, Rome in Italy, Västeras in Sweden, Vienna in Austria and Zaragoza in Spain). The protocol was approved by the appropriate ethics committee in each centre. Written, informed consent was obtained from each subject and both of his/her parents or legal representatives. Participation in the study was voluntary. The sample included a total of 3,865 adolescents recruited through their schools; the latter were randomly selected according to a proportional cluster sampling methodology taking into account geographical repartition within each city, private/public school ratio, and number of classes by school. In order to investigate clinical biochemistry assays and genetic analyses, one third of the classes were randomly selected for blood collection, resulting in a total of 1,155 subjects.

GOLDN

The Genetics of Lipid Lowering Drugs and Diet Network Study was a clinical trial designed to understand genetic factors that influence the response of triglycerides to 1) the lipid-lowering drug fenofibrate and 2) a high fat meal challenge. The study comprised approximately 1,200 individuals from 200 families. In total, 821 participants with diet, genetic, and metabolic data were included in the present analysis.

BPRHS

The Boston Puerto Rican Health Study is a longitudinal cohort designed to examine sociological, environmental, and genetic risk factors for chronic diseases and quality of life in Puerto Rican adults living in the greater Boston, Massachusetts, area. Recruitment occurred from 2004 to 2009. Participants included those who self-identified as Puerto Rican and who were English or Spanish speaking. A total of 1,500 study participants (aged 45-75 y) were recruited at the baseline. The present study was based on 1,312 participants who have complete data available at the baseline for the proposed study.

SWS

The SWS recruited 12,583 non-pregnant women aged 20-34 years, living in the city of Southampton, UK. A total of 3,158 women subsequently gave birth to liveborn singleton infants within the study period. The present analyses involved two groups, the SWS women themselves (n = 1060 in this analysis) and their offspring (n = 2005 in this analysis).

LISA/GINIplus

The influence of Life-style factors on the development of the Immune System and Allergies in East and West Germany (LISA) Study is a population based birth cohort study (1). A total of 3094 healthy, full-term neonates were recruited between 1997 and 1999 in Munich, Leipzig, Wesel and Bad Honnef. The participants were not pre-selected based on family history of allergic diseases.
A total of 5991 mothers and their newborns were recruited into the German Infant study on the influence of Nutrition Intervention PLUS environmental and genetic influences on allergy development (GINIplus) between September 1995 and June 1998 in Munich and Wesel (2). Infants with at least one allergic parent and/or sibling were allocated to the interventional study arm investigating the effect of different hydrolysed formulas for allergy prevention in the first year of life. All children without a family history of allergic diseases and children whose parents did not give consent for the intervention were allocated to the non-interventional arm. Detailed descriptions of the LISAplus and GINIplus studies have been published elsewhere (1,2). DNA was collected at the age 6

© 2019 BIRTH-GENE (BIG) Study Working Group. *JAMA Network Open.*

and 10 years. For the present analysis, only children from the Munich study center were included. For both studies, approval by the local Ethics Committees and written consent from participant's families were obtained.

1. Heinrich, J. et al. Allergens and endotoxin on mothers' mattresses and total immunoglobulin E in cord blood of neonates. Eur Respir J 20, 617-23 (2002).
2. von Berg, A. et al. Impact of early feeding on childhood eczema: development after nutritional intervention compared with the natural course - the GINIplus study up to the age of 6 years. Clin Exp Allergy 40, 627-36 (2010).

SCORM

The Singapore Cohort Of the Risk factors for Myopia (SCORM) was initiated in three schools in Singapore. The total number of eligible students was 2,913. Of the 2,913, children from an eastern school (n = 660) and a northern school (n = 1,023) were invited to participate in November 1999, while children from a western school (n = 1,230) were invited to participate in May 2001. Of the 2,913, 1,979 children aged 7-9 years agreed to participate at baseline (participation rate = 67.9%): children from the eastern school (n = 313), northern school (n = 705) and western school (n = 961). There were four follow-up visits from 2000 to 2005. Of the 1,979 participants, 345 children (17.4%) were lost to follow-up and 1,634 children (82.6%) returned for ocular examination at age 11 years. Of the 1,634 children, we further excluded those that were non-myopic (n = 585), with missing data on age of onset of myopia (n = 14) and those with age of onset of myopia at age 11 (n = 107). Thus, the final sample size for the current analysis was 928 (n = 761 Chinese, n = 113 Malays and n = 54 Indians and others). Informed written consent was obtained after the nature of the study was explained to the parents. The tenets of the Declaration of Helsinki were observed and approved by the Singapore Eye Research Institute Ethnics Committee.

STRIP

The STRIP study is a prospective, randomized lifestyle intervention project that began in 1990-1992 when 1,062 infants aged 5 months were recruited to a dietary intervention trial with the main aim of replacing saturated fat with unsaturated fat in the child's diet. The intervention was continued until the age of 20 years and during the follow-up children's diet and other lifestyle factors, growth and biological risk factors have been closely monitored with repeated measurements.

CLHNS

The Cebu Longitudinal Health and Nutrition Survey (CLHNS) is a community-based birth cohort study that originally enrolled 3,327 pregnant women in 1983-84 (3,080 singleton live births), and has since followed them and their offspring to the present. In 2005, 1,895 healthy Filipino mothers and 1,775 of their offspring remained in the study and from whom DNA and measurement of biomarkers were collected. Trained field staff conducted in-home interviews and collected anthropometric measurements and comprehensive environmental data at each visit (data available online at http://www.cpc.unc.edu/projects/cebu/). Blood samples, which were used for biomarker measurement and DNA extraction, were obtained in 2005. Our analysis included 1,798 CLHNS mothers with both genotype and phenotype data. Informed consent was obtained from all CLHNS participants, and the study protocol was approved by the University of North Carolina Institutional Review Board for the Protection of Human Subjects.

RS

The Rotterdam Study I (RS) is a prospective cohort study ongoing since 1990 in the city of Rotterdam in the Netherlands. All inhabitants aged 55 years and over of the Ommoord district in the city of Rotterdam were invited to participate. In the current analyses we included 6,291 participants for whom we had genetic data available. The participants were all examined at baseline. They were interviewed at home (2 h) and then had an extensive set of examinations (a total of 5 h) in a specially built research facility in the centre of their district. These examinations were repeated every 3-4 years in characteristics that could change over time. The Rotterdam Study has been approved by the institutional review board (Medical Ethics Committee) of Erasmus Medical Center and by the review board of The Netherlands Ministry of Health, Welfare and Sports.

© 2019 BIRTH-GENE (BIG) Study Working Group. *JAMA Network Open.*

NEO

The NEO study was designed for extensive phenotyping to investigate pathways that lead to obesity-related diseases. The NEO study is a population-based, prospective cohort study that includes 6,671 individuals aged 45-65 years, with an oversampling of individuals with overweight or obesity. At baseline, information on demography, lifestyle, and medical history have been collected by questionnaires. In addition, samples of 24-h urine, fasting and postprandial blood plasma and serum, and DNA were collected. Genotyping was performed using the Illumina HumanCoreExome chip, which was subsequently imputed to the 1000 genome reference panal. Participants underwent an extensive physical examination, including anthropometry, electrocardiography, spirometry, and measurement of the carotid artery intima-media thickness by ultrasonography. In random subsamples of participants, magnetic resonance imaging of abdominal fat, pulse wave velocity of the aorta, heart, and brain, magnetic resonance spectroscopy of the liver, indirect calorimetry, dual energy X-ray absorptiometry, or accelerometry measurements were performed. The collection of data started in September 2008 and completed at the end of September 2012. Participants are currently being followed for the incidence of obesity-related diseases and mortality.

CHOP

The Childhood Obesity Project (CHOP) was conducted as a European multicenter, double-blind, randomized clinical trial that enrolled healthy infants born between October 2002 and July 2004. Formula-fed infants (n = 1,090) were randomly assigned to receive higher protein (HP)- or lower protein (LP)-content formula (within recommended amounts) in the first year of life; breastfed infants (n = 588) were enrolled as an observational reference group. Weight, length, weight-for-length, and BMI were determined at inclusion and at 3, 6, 12, and 24 months of age. The primary endpoints were length and weight at 24 months of age. Anthropometric data have been followed since then biannually up to the age of 6 years and at age 8 and 11 years in this ongoing cohort. Comprehensive nutritional, (epi-)genetic and metabolic measurements in the participating children were also collected as well as data on parental and child'smedical history and life-style factors(see: Koletzko B, AJCN 2009; 89:1836-45 (PMID:19386747);Weber M, AJCN 2014;99:1041-51 (PMID:24622805); Rzehak P, Scientific reports 2017;7:14349 (PMID:29084944); Kirchberg FF, J ClinEndocrinolMetab. 2015;100:149-58 (PMID:25368978))

WHI

The Women's Health Initiative (WHI)is a large, multi-centre study designed to study major causes of morbidity and mortality in postmenopausal women. The WHI includes a clinical trial (CT) and an observational study (OS) cohort. Women meeting eligibility criteria (age 50-79, postmenopausal, minimum life expectancy 3 years) were recruited at 40 US clinical centres between 1 September 1993 and 31 December 1998. The study sample included 161,808 participants enrolled in the WHI Observational Study and in the three overlapping clinical trials (hormone therapy, dietary modification, and calcium plus vitamin D) prospectively followed for an average of 12 years or until earliest of treated T2DM, death, loss to follow-up, or end of study. Written informed consent was obtained from all study participants before study enrollment, and each of the trials was approved by the Institutional Review Boards of the 40 participating institutions.

YH3 & YH2000

The Young Hearts (YH) project is a prospective study investigating the development of biological and behavioural risk factors for cardiovascular disease in an adolescent population in Northern Ireland. Briefly, in 1989-1990, a 2% representative sample of school children aged 12 and 15 years in Northern Ireland (YH1, n=1,015) was collected. The original 12-year-old population was followed up in 19921993 (YH2) with complete data collected on 225 boys and 230 girls (90% response rate). Between 1997 and 1999, all original YH participants were invited to participate in the third screening phase (YH3, age 2125 years, n= 489), and a blood sample for DNA extraction was taken at that time. A further cross-sectional survey, the Young Hearts 2000 (YH2000), was carried out in 2000. Approximately 2,000 boys and girls aged 12 and 15 years (500 in each of the four age-sex groups) were recruited through post-primary schools.

© 2019 BIRTH-GENE (BIG) Study Working Group. *JAMA Network Open.*

DF-TJ
The Dongfeng-Tongji Cohort (DF-TJ) was launched in 2008, and conducted by Tongji Medical College, Huazhong University of Science and Technology and Dongfeng Motor Corporation (DMC). There are 27,009 (87% of 31,000) retirees of DMC who responded to a questionnaire and provided baseline blood samples. Our analysis included 1,452 participants whose genotype data were available. All participants in our study provided written informed consent.

HCS
In 1998, a cohort of men and women born 1931-1939 in the county of Hertfordshire, United Kingdom and still resident there, was recruited to participate in studies examining the interactions between early life, diet, adult lifestyle, and genetic factors as determinants of adult disease. A total of 3,225 men and women, aged 59-73 years, were interviewed at home where information was obtained on the participant's medical and social history by a trained research nurse. Subsequently, 2,997 men and women attended a clinic for further investigations.

1958BC
The British 1958 birth cohort (1958BC) includes all births during one week in March in 1958 in England, Scotland and Wales. Approximately 17,000 participants were recruited at birth and were subsequently followed up at ages 7, 11, 16, 23, 33, 42, 45 and 50 years. At each follow-up, information on socioeconomic status, health and development, and familial and education factors were obtained. At 33 and 42 years, diet, lifestyle and occupational factors were also collected. At 45 years of age, 11,971 participants currently living in Britain were invited to take part in a biomedical survey, of whom 9,377 (78%) filled in a questionnaire and 8,302 (89%) also provided a blood sample, in which DNA was extracted for genotyping.

Biobank Japan
The Biobank Japan Project was started in 2003 for aiming at the implementation of personalized medicine as a leading project of MEXT, Japanese government. This project developed a disease (patient-registered) biobank of 47 common diseases in collaboration with 12 cooperating institutes and collected a total of 200,000 patients' DNA and clinical information. Various clinical information was collected from interview or medical records by using standardized questionnaire. All participants gave written informed consent to this study. This study was approved by ethical committees of RIKEN and participating institutes.

LURIC
The LURIC study included 3,316 Caucasians hospitalized for coronary angiography between 1997 and 2000 at a tertiary care centre in south-western Germany. Clinical indications for angiography were chest pain or a positive non-invasive stress test suggestive of myocardial ischemia. To limit clinical heterogeneity, individuals suffering from acute illnesses other than acute coronary syndrome, chronic non-cardiac diseases and a history of malignancy within the five past years were excluded. The study was approved by the ethics committee at the "LandesärztekammerRheinland-Pfalz" and was conducted in accordance with the "Declaration of Helsinki". Informed written consent was obtained from all participants.

ARIC
ARIC is a multi-center prospective investigation of atherosclerotic disease in a predominantly bi-racial population conducted in four U.S. communities, involving both cohort and community surveillance components. Study participants aged 45-64 years at baseline were recruited from 4 communities: Forsyth County, North Carolina; Jackson, Mississippi; suburban areas of Minneapolis, Minnesota; and Washington County, Maryland. A total of 15,792 individuals participated in the baseline examination in 1987-1989, with follow-up examinations in approximate 3-year intervals, during 1990-1992, 1993-1995, and 1996-1998.Weight and height were measured. All study participants provided written informed consent.

© 2019 BIRTH-GENE (BIG) Study Working Group. *JAMA Network Open.*

Bogalusa Study
The Bogalusa Heart Study is a community-based study of the natural history of cardiovascular disease since childhood in the community of Bogalusa, Louisiana. The study is longitudinal, and the initial cross-sectional study began in 1973-1974. Subsequent cross-sectional surveys were conducted every 34 years during childhood and young adulthood. Our analysis included 619 subjects whose genotype and birth weight data were available. Information on personal health and medication history was obtained from participants through questionnaires. Standard protocols approved by the Institutional Review Board of the Tulane University Health Sciences Center were used for the collection of all data. Written informed consent was obtained from the participants, or their parents (guardians) if the participants were children.

SiMES
Singapore Malay Eye Study (SiMES) was conducted at the Singapore Eye Research Institute(SERI) and designed to quantify the prevalence of and risk factors for visual impairment and major eye diseases, including refractive errors, glaucoma, cataract, diabetic retinopathy, and age-related maculopathy, in an adult urban Malay population in Singapore.

SINDI
Singapore Indian Eye Study (SINDI) was designed to quantify the prevalence, environmental and genetic risk factors, and impacts of visual impairment and major eye diseases in Singapore Indian population.

DC-SP2 (SP2-SDCS-610 and SP2-SDCS-1M)
Diabetic Cohort (DC) was designed to study the prevalence of diabetes in Singapore Chinese population.

SCES
Singapore Chinese Eye Study (SCES) was designed to quantify the prevalence, environmental and genetic risk factors, and impacts of visual impairment and major eye diseases in Singapore Chinese population.

CHS
The CHS is a population-based longitudinal study of risk factors for cardiovascular disease and stroke in adults 65 years of age or older, recruited at four field centers (Forsyth County, NC; Sacramento County, CA; Washington County, MD; Pittsburgh, PA) . Overall, 5,201 predominantly Caucasian individuals were recruited in 1989-1990 from random samples of Medicare eligibility lists, followed by an additional 687 African-Americans recruited in 1992-1993 (n=5,888). The CHS GWAS, which had the primary aim of studying incident cardiovascular events, focused on 3,980 participants free of clinical cardiovascular disease at baseline, who consented to genetic testing, and with DNA available for genotyping. CHS Study samples were genotyped using the Illumina HumanCNV370-Duo BeadChip system. Participants of European descent were included in this analysis. Genotyping was successful in 3,291 Caucasian subjects. Participants were eligible for the present investigation if their genotyping was complete and they had available phenotype information. Samples with call rate <95% were excluded. A total of 306,655 autosomal SNPs were used in imputation after filtering out SNPs with HWE deviation $P \leq 1 \times 10^{-5}$, call frequency ≤97%, zero heterozygote frequency, missing from dbSNP, and >1 duplicate or Mendelian inconsistency.

SCHS (SCHS-T2D)
This study utilized data from a case-control GWAS study for T2DM that was nested within the Singapore Chinese Health Study (SCHS), a prospective cohort study of 63,257 Singaporean Chinese men and women aged 45-74 years living in Singapore between 1993 and 1998. The cohort study recruited only participants belonging to one of the two major Chinese dialect groups in Singapore, the Hokkiens or the Cantonese, who originated from two contiguous prefectures in southern China. In-person interviews and phlebotomy were conducted before the onset of disease. Baseline assessment was conducted through a face-to-face structured interview during recruitment. Information was

© 2019 BIRTH-GENE (BIG) Study Working Group. *JAMA Network Open.*

recorded by a trained interviewer using a structured questionnaire. Food frequency questionnaires were used to estimate usual diet among the SCHS participants and daily proportions of macronutrients intake. Starting in January 2000, the bio-specimen collection was extended to all surviving cohort members. By April 2005 when all subjects had been contacted, bio-specimens were collected from 32,543 participants, representing an approximately 60% consent rate. Various components (plasma, red blood cell, serum and white blood cell) of blood were separated and have been stored continuously at -80°C. Age was defined at the time of blood collection and sex and ethnicity was recorded as indicated in the participant's National Registration Identity Card (NRIC). Informed consent was obtained from all subjects and approval by the Institutional Review Board of the National University of Singapore was given for this study.

© 2019 BIRTH-GENE (BIG) Study Working Group. *JAMA Network Open.*

eReferences

1. Horikoshi M, Yaghootkar H, Mook-Kanamori DO, et al. New loci associated with birth weight identify genetic links between intrauterine growth and adult height and metabolism. *Nature genetics.* Jan 2013;45(1):76-82.
2. Higgins JP, Thompson SG, Deeks JJ, Altman DG. Measuring inconsistency in meta-analyses. *Bmj.* Sep 6 2003;327(7414):557-560.
3. Ioannidis JP, Patsopoulos NA, Evangelou E. Uncertainty in heterogeneity estimates in meta-analyses. *Bmj.* Nov 3 2007;335(7626):914-916.
4. Higgins JP, Thompson SG. Quantifying heterogeneity in a meta-analysis. *Stat Med.* Jun 15 2002;21(11):1539-1558.
5. Riley RD, Higgins JP, Deeks JJ. Interpretation of random effects meta-analyses. *Bmj.* 2011;342:d549.
6. Wald A. The fitting of straight lines if both variables are subject to error. *Ann Math Stat.* 1940;11:284–300.
7. Palmer TM, Sterne JA, Harbord RM, et al. Instrumental variable estimation of causal risk ratios and causal odds ratios in Mendelian randomization analyses. *Am J Epidemiol.* Jun 15 2011;173(12):1392-1403.
8. Horikoshi M, Beaumont RN, Day FR, et al. Genome-wide associations for birth weight and correlations with adult disease. *Nature.* Oct 13 2016;538(7624):248-252.
9. Mahajan A, Go MJ, Zhang W, et al. Genome-wide trans-ancestry meta-analysis provides insight into the genetic architecture of T2DM susceptibility. *Nature genetics.* Mar 2014;46(3):234-244.
10. Lawlor DA, Harbord RM, Sterne JA, Timpson N, Davey Smith G. Mendelian randomization: using genes as instruments for making causal inferences in epidemiology. *Statistics in medicine.* Apr 15 2008;27(8):1133-1163.
11. Burgess S, Scott RA, Timpson NJ, Davey Smith G, Thompson SG. Using published data in Mendelian randomization: a blueprint for efficient identification of causal risk factors. *European journal of epidemiology.* Jul 2015;30(7):543-552.
12. Burgess S, Butterworth A, Thompson SG. Mendelian randomization analysis with multiple genetic variants using summarized data. *Genetic epidemiology.* Nov 2013;37(7):658-665.
13. Bowden J, Davey Smith G, Burgess S. Mendelian randomization with invalid instruments: effect estimation and bias detection through Egger regression. *International journal of epidemiology.* Apr 2015;44(2):512-525.
14. Bowden J, Davey Smith G, Haycock PC, Burgess S. Consistent Estimation in Mendelian Randomization with Some Invalid Instruments Using a Weighted Median Estimator. *Genetic epidemiology.* May 2016;40(4):304-314.
15. Yavorska OO, Burgess S. MendelianRandomization: an R package for performing Mendelian randomization analyses using summarized data. *International journal of epidemiology.* Apr 07 2017.
16. Burgess S, Bowden J, Fall T, Ingelsson E, Thompson SG. Sensitivity Analyses for Robust Causal Inference from Mendelian Randomization Analyses with Multiple Genetic Variants. *Epidemiology.* Jan 2017;28(1):30-42.
17. Scott RA, Lagou V, Welch RP, et al. Large-scale association analyses identify new loci influencing glycemic traits and provide insight into the underlying biological pathways. *Nature genetics.* Sep 2012;44(9):991-1005.
18. Emdin CA, Khera AV, Natarajan P, et al. Genetic Association of Waist-to-Hip Ratio With Cardiometabolic Traits, T2DM, and Coronary Heart Disease. *Jama.* Feb 14 2017;317(6):626-634.
19. Centers for Disease Control and Prevention. National diabetes statistics report: estimates of diabetes and its burden in the United States, 2014. US Department of Health and Human Services. 2014. .

© 2019 BIRTH-GENE (BIG) Study Working Group. *JAMA Network Open.*

© 2019 BIRTH-GENE (BIG) Study Working Group. *JAMA Network Open.*
